# Posttranslational modifications optimize the ability of SARS-CoV-2 spike for effective interaction with host cell receptors

**DOI:** 10.1073/pnas.2119761119

**Published:** 2022-06-23

**Authors:** Karan Kapoor, Tianle Chen, Emad Tajkhorshid

**Affiliations:** ^a^Theoretical and Computational Biophysics Group, NIH Center for Macromolecular Modeling and Bioinformatics, Beckman Institute for Advanced Science and Technology, Department of Biochemistry, University of Illinois at Urbana–Champaign, Urbana, IL 61801;; ^b^Center for Biophysics and Quantitative Biology, University of Illinois at Urbana Champaign, Urbana, IL 61801

**Keywords:** spike protein, coronaviruses, structural dynamics, glycosylation, molecular dynamics

## Abstract

Severe acute respiratory syndrome coronavirus 2 (SARS-CoV-2) spike glycoprotein is the prime target for vaccines, diagnostics, and therapeutic antibodies against the virus. While anchored in the viral envelope, for effective virulence, the spike needs to maintain structural flexibility to recognize the host cell surface receptors and bind to them, a property that can heavily depend upon the dynamics of the unresolved domains, most prominently the stalk. Construction of the complete, membrane-bound spike model and the description of its dynamics are critical steps in understanding the inner working of this key element of the viral infection by SARS-CoV-2. Combining homology modeling, protein–protein docking, and molecular dynamics (MD) simulations, we have developed a full spike structure in a native membrane. Multimicrosecond MD simulations of this model, the longest known single trajectory of the full spike, reveal conformational dynamics employed by the protein to explore the surface of the host cell. In agreement with cryogenic electron microscopy (cryo-EM), three flexible hinges in the stalk allow for global conformational heterogeneity of spike in the fully glycosylated system mediated by glycan–glycan and glycan–lipid interactions. The dynamical range of the spike is considerably reduced in its nonglycosylated form, confining the area explored by the spike on the host cell surface. Furthermore, palmitoylation of the membrane domain amplifies the local curvature that may prime the fusion. We show that the identified hinge regions are highly conserved in SARS coronaviruses, highlighting their functional importance in enhancing viral infection, and thereby, provide points for discovery of alternative therapeutics against the virus.

## Significance

SARS-CoV-2 spike protein, which forms the basis for high pathogenicity and transmissibility of the virus, is a prime target for the development of both diagnostics and vaccines for the debilitating disease caused by the virus. We present a full model of spike methodically crafted and used to study its atomic-level dynamics by multiple microsecond simulations. The results shed light on the impact of posttranslational modifications on the pathogenicity of the virus. We show how glycan–glycan and glycan–lipid interactions broaden the protein’s dynamical range and thereby, its effective interaction with the surface receptors on the host cell. Palmitoylation of the spike membrane domain, however, results in a unique deformation pattern that might prime the membrane for fusion.

The pandemic caused by severe acute respiratory syndrome coronavirus 2 (SARS-CoV-2) continues to impact nearly all aspects of human life (1, 2). SARS-CoV-2 belongs to the beta coronavirus genus, the same as SARS-CoV and Middle East respiratory syndrome coronavirus (MERS-CoV), which were responsible for the severe acute respiratory syndrome outbreak in 2003 and the Middle East respiratory syndrome in 2012, respectively (3, 4). The first step in viral infection by SARS-CoV-2 and other coronaviruses in general is the binding of its extended spike glycoproteins to specific human surface receptors (5, 6). Spike proteins contain all components necessary for viral entry into human cells; after binding to the surface receptors, they initiate the fusion of the viral and human membranes and subsequent release of the viral genome into the host cell (7). Characterizing the mechanism of action of the spike protein thus remains key to our understanding of the critical steps in viral infection, paving the way for development of therapeutic strategies against the virus.

**Fig. 1.**
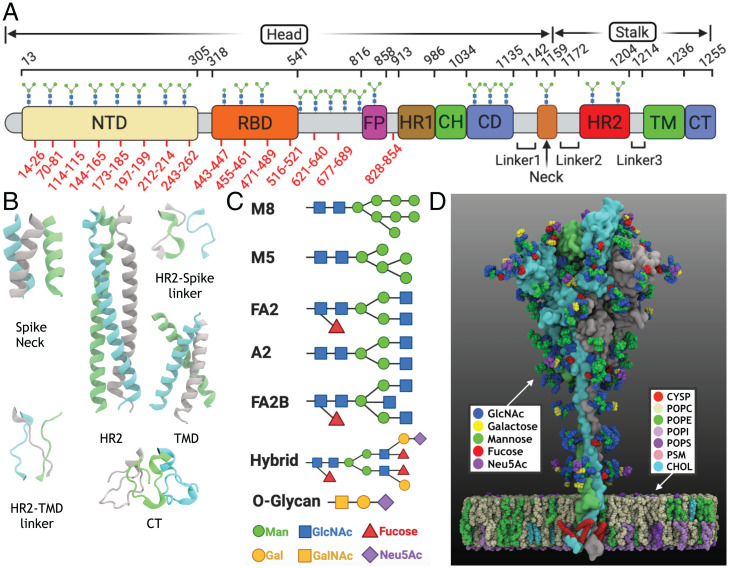
Overview of the full spike system. (*A*) Schematic of the different functional domains of the spike protein, which can be largely divided into the head and stalk domains: N-terminal domain (NTD), RBD, fusion peptide (FP), heptad repeat 1 (HR1), central helix (CH), connecting domain (CD), spike neck, HR2, TM, and cytoplasmic tail (CT). The residue ranges of these domains are provided along with the experimentally identified glycosylation sites marked on top of the domains. Of these, cryo-EM structures are only available for the spike head, with the missing regions in the structure shown in red. (*B*) The modeled structures in the spike neck, HR2, TM domain, CT, and the two connecting linkers are shown. These missing regions in the spike stalk were modeled using a hybrid approach (*Materials and Methods*). (*C*) The types of *N*-linked and *O*-linked glycans modeled in the full spike structure are shown. (*D*) The modeled structure of the full-length, palmitoylated, and glycosylated spike is shown. Each spike monomer is depicted in surface representation in a different color. The protein is embedded in a membrane with the lipid composition of ERGIC. The lipid composition is: 1-palmitoyl-2-oleoyl-sn-glycero-3-phosphocholine (POPC), 1-palmitoyl-2-oleoyl-sn-glycero-3-phosphoethanolamine (POPE), 1-palmitoyl-2-oleoyl-sn-glycero-3-phosphoinositol (POPI), 1-palmitoyl-2-oleoyl-sn-glycero-3-phospho-L-serine (POPS), palmitoylsphingomyelin (PSM), cholesterol (CHOL), and palmitoylated cysteine (CYSP). The sugar types in the glycosylations are: galactose, mannose, fucose, N-acetyl-D-glucosamine (glcNAc) and N-acetyl-D-neuraminic acid (Neu5Ac).

Several SARS-CoV-2 spike cryo-EM structures have been resolved (8–15). Although providing essential information on the structural details of the spike globular domain (spike head), which contains the receptor-binding domain (RBD) binding the Angiotensin-converting enzyme 2 (ACE2) host cell receptors (6), the structures still lack functionally important regions (Fig. 1*A*). These include the conserved fusion peptide involved in initiating the fusion (16, 17), the stalk heptad repeat 2 (HR2) domain (18), and the transmembrane (TM) domain containing multiple palmitoylation sites that stabilize the spike in the envelope and thus, are essential for its assembly and activity in other viruses (19, 20). The structures also lack critical information about the glycosylation of different residues, known to be essential in mediating protein folding and shaping viral tropism as well as shielding the virus from immune recognition (21).

Additionally, the assembly and budding of the virus are known to take place in the endoplasmic reticulum–Golgi intermediate compartment (ERGIC) (22, 23), an environment that is often not taken into account in structural studies.

Computational simulations offer an effective technique for modeling complex systems like the spike at a detailed level. Recent classical molecular dynamics (MD) simulations of the spike head (24–29) as well as constructed full-length spike models (30–32) have provided powerful insights into functionally relevant features [e.g., how glycans can shield the spike from the immune system (24, 25, 28, 31, 32) or the intradomain dynamics of the spike head that may facilitate the RBD to switch between the “up” and “down” (active or inactive) conformations (26–29, 31)]. However, we are still far from fully understanding how the global dynamics of the spike may contribute to its function, specifically in relation to effectively locating its target receptor in crowded cellular environments. In this context, characterizing how glycosylation may play a role in the protein’s global conformational dynamics is as important. Furthermore, the role of palmitoylations in the TM/endodomain, which are known to participate in the modulation of the membrane curvature and in mediating cell fusion in other viruses (33–36), remains lacking in SARS-CoV-2.

We constructed a full-length, membrane-embedded spike and investigated its dynamics by atomistic MD simulations. Taking a hybrid approach combining homology modeling, protein–protein docking and MD, and the available experimental data (37–40), a full-length, palmitoylated, and fully glycosylated spike is modeled. After validating the modeled parts, multimicrosecond MD simulations of the full spike were used to characterize its global dynamics, focusing particularly on the role of glycans. Control simulations of the nonglycosyslated spike highlighted the importance of the glycans in the global motion of the protein. Palmitoylation of the protein is found to modulate the membrane curvature, potentially facilitating its fusion with the host cell. This study provides insight into the dynamics used by the spike to sample the surface of its host cell and the direct role of the glycans in regulating this process while successfully allowing the virus to evade the host immune response.

## Results and Discussion

Here, we first develop a full spike structure in its membrane-bound state and use it for microsecond-scale MD simulations to characterize its conformational dynamics. We describe these conformational changes in terms of bending and twisting motions of the spike head with respect to the stalk and examine the potential role of glycan–lipid and glycan–glycan interactions in these motions. Sequence comparison with other human coronaviruses allows us to delineate multiple conserved hinge regions in the stalk that likely promote the conformational motions of the spike head. Furthermore, we quantify the effects of palmitoylation in the TM domain, offering support for the role of this posttranslational modification in modulating the local membrane curvature.

### Construction of the Membrane-Embedded Full Spike Protein.

We combined homology modeling utilizing multiple template structures from homologous coronaviruses, like SARS-CoV and MERS-CoV, with fragment-based, ab initio modeling and secondary structure prediction to construct the missing regions in the spike head and the stalk (Fig. 1 *A* and *B* and *SI Appendix*, Fig. S1). Additionally, we carried out an expansive procedure for deriving a stable model of the TM domain in a native membrane (*SI Appendix*, Table S1), where we combined sequence-based secondary structure prediction for the TM region (*SI Appendix*, Fig. S2) with protein–protein docking for generating the initial configurations of TM trimers (*SI Appendix*, Table S3) followed by MD relaxation of suitable TM trimeric configurations in membrane (*SI Appendix*, Figs. S3 and S4). The above-described modeling procedure was combined with the biochemical data on the palmitoylation sites (37) and the recently reported glycomics data to determine the glycosylation compositions of specific Asn residues (38, 39) (Fig. 1*C* and *SI Appendix*, Table S2) to develop the full-length, palmitoylated, and fully glycosylated spike structure (Fig. 1*D*).

**Fig. 2.**
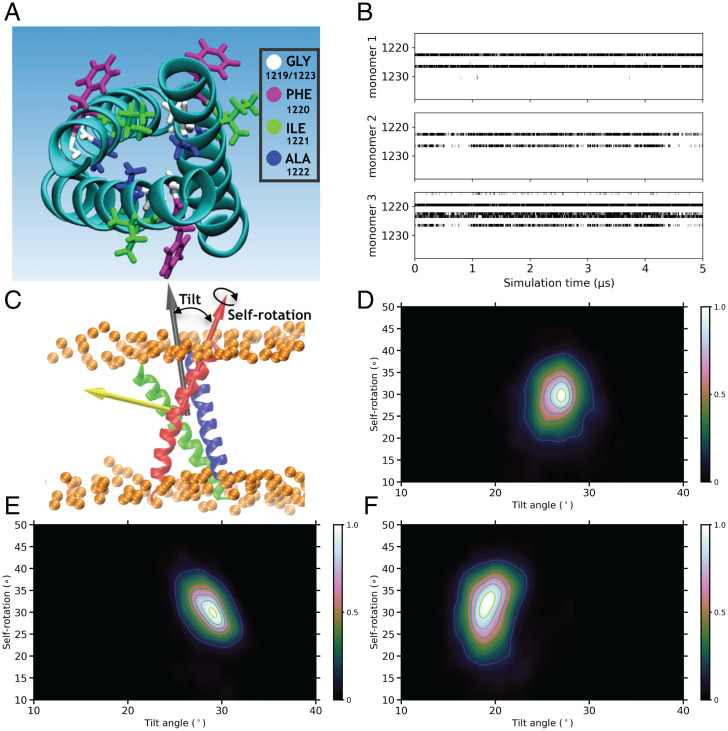
Stability of the modeled TM domain. (*A*) The top view of the modeled TM domain and the licorice representation of the GxxxG motif (residues 1,219 to 1,223) are shown. (*B*) Contact maps between a TM monomer and the other two monomers in the TM domain during the trajectory. A black dot is used to indicate the presence of at least one C${}_{\alpha }$–C${}_{\alpha }$ contact (4.0-Å cutoff) between residues of a monomer and the other two monomers. (*C*) TM monomers’ self-rotation and tilt motions. The self-rotation of each monomer is calculated with respect to its equilibrated configuration, and the tilt angle is measured with respect to the whole TM domain. Heat maps for the distribution of the tilt angle vs. the self-rotation angle are shown for the TM monomer 1 (*D*), monomer 2 (*E*), and monomer 3 (*F*).

We have also carried out structural comparison of our full-length spike model with previously published models (30, 31, 41). The individual monomers of the spike head display relatively stable conformations after the initial equilibration (*SI Appendix*, Fig. S5). The HR2 domain in the previous studies was modeled using a coiled-coil template from *Salmonella* autotransporter adhesin (31), using a de novo coiled-coil modeling program (41), or using the HR2 from SARS-CoV (30). Here, we also make use of the HR2 template from SARS-CoV (42), resulting in a stable model throughout the simulations (*SI Appendix*, Fig. S5). As SARS-CoV and SARS-CoV-2 display complete sequence identity in the HR2 domain, our stable model is expected to provide a closer physiological representation of this domain. The TM domain in the previous spike models (30, 31) was constructed using a template structure from HIV (43). It is noteworthy that the TM helices in HIV each contain a “GxxxG” motif, known to be involved in inter-TM helix interactions (44). Consequently, the TM domain assembly in HIV displays interhelical contacts in this region with the C-terminal half only held together by polar contacts (43). As the GxxxG motif is present in the central region of the TM helices in the SARS-CoV-2 spike (Fig. 1*A*), its contacts between the TM helices can be expected to lie in this region instead. The TM domain in our spike model is a trimeric assembly, with the majority of the interhelical contacts formed between the central regions of the helices containing the GxxxG motifs (Fig. 2 *A* and *B*). Additionally, the TM domain maintains a relatively stable and symmetric structure during the simulations (Fig. 2 *C*–*F* and *SI Appendix*, Fig. S5). The correct arrangement of the different stalk domain structures, including the trimeric HR2 and TM domains, can be critical to the correct description of the spike’s global dynamics.

### Bending and Twisting Motions of the Spike Head around the Stalk.

**Fig. 3.**
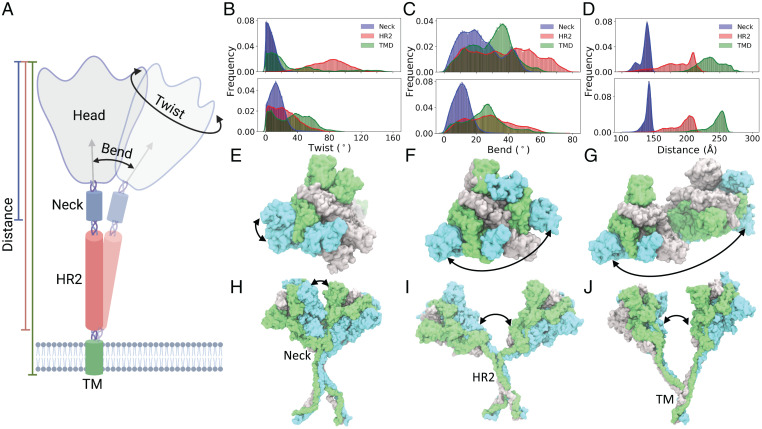
Global conformational dynamics of spike. (*A*) The schematic depicts the calculated bend and twist motions of the spike head (gray) around the spike neck (blue), the HR2 domain (red), and the TM domain (green) as well as the calculated head distances with respect to these domains. The probability distributions (in terms of normalized frequency) of the (*B*) twist, (*C*) bend, and (*D*) distance of the spike head with respect to the spike neck, HR2 domain, and TM domain are provided. *Upper* and *Lower* show the distributions observed in the fully glycosylated spike and the nonglycosylated spike, respectively. Example snapshots of the spike (surface representation with each monomer in a different color) displaying the largest head twist with respect to (*E*) the spike neck, (*F*) the HR2 domain, and (*G*) the TM domain are shown for the fully glycosylated system. Similarly, example snapshots of the spike showing the largest head bend with respect to (*H*) the spike neck, (*I*) the HR2 domain, and (*J*) TM domain are shown.

To further describe the dynamics employed by the spike to sample the crowded cellular surface, we characterized its global motions in terms of the movements of the spike head with respect to the stalk region during the simulations (Movie S1). These motions were quantified in terms of head-twist and head-bend angles as well as head distances calculated separately with respect to the spike neck, the HR2 domain, or the TM domain (Fig. 3*A*). In its fully glycosylated, native form, the spike can sample large head-twist angles (in the *xy* plane) with respect to both the HR2 and TM domains, highlighting the large conformational flexibility available along this degree of freedom (Fig. 3 *B*, *Upper*, *F*, and *G*). A wide normal-like distribution of head-twist angles is observed with respect to the HR2 domain, with the highest sampled configurations turned by almost $90^{{}^{\circ}}$ relative to the starting structure. With respect to the TM domain, although lowly populated, the spike head can sample conformations that are almost oppositely facing (turned by as much as $160^{{}^{\circ}}$) compared with the starting orientation. A relatively smaller range of motion is allowed around the spike neck, with the most populous regions lying close to the starting structure (Fig. 3 *B*, *Upper* and *E*). These results can be directly related to the length of the linkers connecting the different domains of spike. The domains around which the spike head displays larger motions (HR2 and TM domains) are connected by longer linkers (linker 2: 13 residues; linker 3: 11 residues) compared with the domain around which the spike head displays relatively more restricted motions (spike neck), which are connected to the spike head by a smaller linker (linker 1: 7 residues). Furthermore, direct comparison of the head-twist motions in the glycosylated form with the control nonglycosylated system shows surprisingly that these motions become restricted in the latter (Fig. 3 *B*, *Lower*), especially with respect to the head-twist angles calculated around the HR2 and TM domains. These results point to an unexpected enhancing role of glycans in the overall dynamics of the spike.

Similarly, the spike head displays a broad range of head-bend angles with respect to the HR2 domain, sampling configurations almost orthogonal to the starting structure (Fig. 3 *C*, *Upper* and *I*). Comparatively, smaller head-bend motions are observed around the spike neck and the TM domain, with the respective populations showing distributions over a range of 0^∘^ to 60^∘^ angles (Fig. 3 *C*, *Upper*, *H*, and *J*). The extent of these bending angles is also directly related to the changes in the head distances with respect to these domains, with the largest distance changes observed in conformations with the largest head-bend angles (Fig. 3 *D*, *Upper*). Thus, the spike head can move as much as 125 Å with respect to the HR2 domain compared with its starting configuration. Again, comparison of the glycosylated and nonglycosylated forms shows that the spike domains rigidify (display a relatively smaller range of motion) in the absence of glycans (Fig. 3 *C* and *D*, *Lower*). The potential role of the glycans in modulating global motions of the spike is further discussed in later sections.

Mechanistically, these motions (bend/twist/translation) of the spike head represent major degrees of freedom for the RBD (located in the spike head) to locate and bind to the target ACE2 receptors in the host cell membranes. As spike proteins have been found to be sparsely distributed on the virion envelope [recent cryo-EM and tomography studies (12, 45)], the global flexibility of the spike head and its allowed range of motions may play an important role in expanding the search space covered by the spikes. Cryo-EM and previous computational studies have similarly reported the conformational diversity of the spike head around its stalk (41, 46). Compared with our results, a smaller conformational variability of the spike head was observed by Turoňová et al. (41); as stated by the authors, the crowded conditions generated by inclusion of four spike proteins in the simulation system might have prevented ample sampling of the available space. The crowded distribution used in that study does not represent the physiological density reported for the full virion in later cryo-EM studies (approximately one spike per 1,000 nm^2^ of the viral surface area) (45). While previous simulation studies on the spike protein have concentrated on the interdomain motions in the protein (41, 46) or on the conformational dynamics of the RBD (open/closed transitions) (27–29) and its response to glycosylation (28, 31), in the present study we focus more on how glycans may modulate the global motions of the entire spike, particularly with respect to the positioning of the head domain with respect to the viral envelope (discussed in later sections).

### Sequence Conservation in Multiple Hinge Regions.

**Fig. 4.**
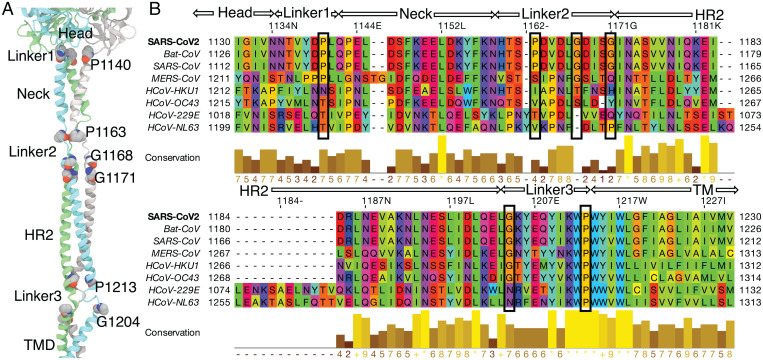
Multiple sequence alignment of spike homologs. (*A*) The structure of the stalk region of the spike in our model is shown. Pro and Gly residues of the linkers are shown in van der Waals representation. (*B*) The sequence of the SARS-CoV-2 spike is aligned with those from closely related viruses (bat coronavirus RaTG13 and six other human CoV viruses). The residues are colored according to their physicochemical properties in different shades of rainbow colors (Taylor coloring scheme). The different domains and linkers are labeled at the top of the sequence. The completely and partially conserved Pro and Gly residues present in the flexible linkers are enclosed in black boxes. Conservation between the different sequences is visualized as histograms with their heights and color variation from brown to yellow, reflecting the level of conservation of physicochemical properties. Conserved and identical amino acid columns show the highest score of 11 and are indicated by *, whereas conserved and similar amino acid columns show a score of 10 and are indicated by +. Other groupings are indicated by lower scores accordingly.

In order to examine potential generality of the observed global motions in the SARS-CoV-2 spike, we analyzed the sequence similarity of the spike protein, specifically focusing on the flexible regions connecting the different domains. These include the small connecting linkers between the spike head and the neck (linker 1: NTVYDPL), between the spike neck and the HR2 domain (linker 2: HTSPDVDLGDISG), and between the HR2 domain and the TM domain (linker 3: GKYEQYIKWP). The three linkers are found to be rich in Pro and Gly, residues known to contribute to the conformational flexibility in proteins (47, 48) (Fig. 4*A*). Additionally, the Pro residues in these regions may be important for the hinge or recoil motion suggested to take place in the refolding of the spike during the fusion process, which may result in an elongated postfusion state of the protein (15).

To further elucidate the possible functional importance of these residues in the mobility of the spike, multiple sequence alignment was carried out with other coronaviruses infecting humans. Interestingly, all Pro and Gly residues present in the flexible regions are completely conserved in the homologous bat-CoV and human SARS-CoV and to a large extent, in human MERS-CoV, where only Pro-1163 is not conserved (Fig. 4*B*). Most of these residues are not conserved in other human coronaviruses except Pro-1213, which is completely conserved in all coronaviruses. We propose that the presence of these conserved flexible residues in the regions connecting the different domains not only promotes the observed conformational flexibility of the spike head around the stalk domains but compared with other coronaviruses, also provides an evolutionary advantage to SARS-CoV-2, SARS-CoV, and MERS-CoV, coronaviruses known to be highly infective in humans.

High glycan densities are also observed around both these flexible regions and the stalk overall in addition to the high densities observed around the spike head, providing a global shielding mechanism for the spike against the immune response and neutralizing antibodies (*SI Appendix*, Fig. S6*A*). The accessibility of the spike protein, measured independently for the glycosylated and nonglycosylated systems, is found to increase monotonically with increasing probe radius (*SI Appendix*, Fig. S6*B*), consistent with the previous MD results (31). In addition to the local, residue-wise profile of glycan shielding (28, 32), our calculation of the global shielding effects examined here provides a more global view of how glycosylation may prevent antibodies targeting the spike.

The spike protein is composed of two subunits: S1, which contains the RBD that recognizes and binds to the human ACE2 (hACE2) receptor, and S2, which includes the stalk domain along with the fusion machinery that mediates the viral membrane fusion after cleavage and separation from the S1 subunit. Recent studies have shown that antibodies targeting the S2 subunit exhibit specific neutralizing activity against SARS-CoV-2 (49). Analyzing clinical samples with virus-reactive memory B cells suggests the presence of circulating immunoglobulin G antibodies reactive to the S2 subunit (50). Furthermore, in silico docking studies have also predicted binding of naturally occurring compounds like flavonoids to the S2 subunit (51). Even though direct evidence for the involvement of the stalk domain and the Pro- and Gly-rich regions of the S2 subunit in interacting with above-mentioned antibodies/small molecules is still lacking, the dynamics of the hinge regions seem to be a highly relevant feature of the protein interacting with other molecules and may provide an additional strategy in developing therapeutics against the virus.

### Stalk Bending Motions Modulated by Glycan–Lipid and Glycan–Glycan Interactions.

The extensive glycosylation of the spike surface is key to the viral escaping from the immune system (28, 31, 32, 41, 46). Moreover, the RBD opening/closing transition, which has been proposed to equip the virus with a “conformational masking” mechanism to hide active RBD from the immune system (28), may be affected by the glycans. Sztain et al. (29) performed over 130 µs of weighted ensemble simulations to reveal a gating role of the glycans on the RBD, and Zimmerman et al. (28) identified cryptic epitopes by capturing dramatic RBD openings calculated for both glycosylated and unglycosylated spikes. However, the underlying mechanism for the dynamics of its stalk region is still undercharacterized. Using our sampling, we have explored how glycans near the hinge regions may modulate their flexibility and affect the global conformation of the spike. We also find that the mobility of the spike stalk would be altered in the absence of glycosylation in our control simulation.

**Fig. 5.**
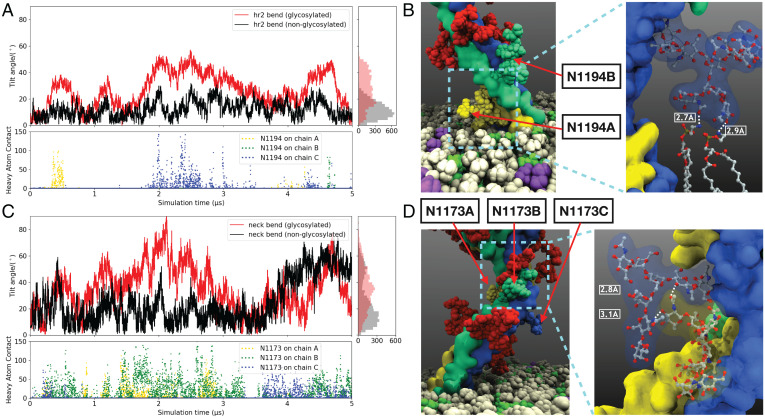
Glycan–glycan and glycan–lipid interactions: (*A*) Time series of the HR2 tilt angle toward the membrane in the glycosylated (red) and nonglycosylated (black) simulations (distribution histograms are shown in *Right*). The numbers of heavy-atom contacts (within 4.5 Å) between the glycans at N1194 and lipids over the course of the trajectory are also shown (*Lower*). The contacts shown use the same color scheme as the corresponding monomer and glycan representations shown in *B* and *D*. (*B*) Example of an N1194 glycan contacting lipids in van der Waals representation, with *Inset* showing the molecular details in CPK representation. The hydrogen bonds between the glycan and lipids are shown as dashed lines in *Inset*. (*C*) Similar tilt angle between the HR2 domain and the neck region of the glycosylated (red) and nonglycosylated (black) simulations along with the distribution histogram (*Right*). The numbers of heavy-atom contacts between the N1173 glycans (HR2) and the N1158 glycans (neck region) are shown as points (*Lower*). (*D*) Example of an N1173 glycan (same color scheme as chains) contacting glycans at N1168 (red) in van der Waals (vdW) representation, with *Inset* showing the molecular details in Corey-Pauling-Koltun (CPK) representation. The hydrogen bonds between the two glycans are marked with dashed lines.

We noted that the glycans also form contacts with each other, as well as with the lipid bilayer, during the simulations (examples of these phenomena are in Movies S2 and S3). To obtain deeper insights into the impact of these glycan interactions, especially with respect to the spike global motions, we evaluated the relationship between the inclination of the different stalk domains and the glycan–lipid or glycan–glycan contacts formed both in the native glycosylated spike simulations and in the control nonglycosylated spike. In the glycosylated spike when the inclination angle of the HR2 domain exceeded a threshold of around $20^{{}^{\circ}}$, we observed formation of more glycan–lipid or glycan–glycan contacts, indicating a correlation between the bending of the HR2 domain and the glycan interactions (Fig. 5). On examining the distribution of the HR2 tilt angles toward the membrane in the glycosylated spike simulation, only ∼20% of the trajectory was found to lie between $0^{{}^{\circ}}$ and $20^{{}^{\circ}}$ (vertical orientation) compared with ∼80% of the nonglycosylated trajectory that was below the $20^{{}^{\circ}}$ inclination. It can be inferred that in the fully glycosylated spike when no glycan–lipid interactions are formed, the HR2 domain can move freely within the $20^{{}^{\circ}}$ inclination (Fig. 5 *A* and *B*); the glycan–lipid interactions appear when the inclination angle is between $20^{{}^{\circ}}$ and $50^{{}^{\circ}}$, further restraining the flexibility of the HR2 hinge motion in this range. A Spearman’s coefficient of 0.59 ±0.08 between the glycan–lipid contacts and HR2 bending further supports the observed correlation.

The HR2 neck inclination shows similar properties as the HR2 membrane inclination, where larger inclination angles, ranging between $20^{{}^{\circ}}$ and $90^{{}^{\circ}}$, mostly correspond with high glycan–glycan contacts (Fig. 5 *C* and *D*). Although the HR2 domain in the nonglycosylated spike can show a similar range of motion (with respect to the neck) as in the native form, almost half of the sampled configurations are close to the starting vertical orientation ($0^{{}^{\circ}}$ and $20^{{}^{\circ}}$) (Fig. 5 *A* and *C*), suggesting coupling between glycan–glycan interactions and the dynamics of the stalk. A moderate Spearman’s coefficient of 0.45 ±0.08 between the glycan–glycan contacts and neck bending provides further support for this observation. Overall, the data indicate that the glycans around the flexible linkers can further enhance the relative motion between the stalk domains, in turn increasing the probed space by the spike head.

*N*-glycans have been reported to be involved in mediating receptor recognition and facilitating cell adhesion (52). The self-recognition and conjugation have been previously demonstrated for *N*-linked complex glycans with multiple *N*-acetylglucosamine (GlcNAc) termini (53, 54). The glycans located at the bottom end of the neck region (N1158) as well as the top (N1173) and bottom (N1194) ends of the HR2 domain are all complex glycans containing multivalent GlcNAc (*SI Appendix*, Table S2). Additionally, these glycans are completely conserved in human coronaviruses (Fig. 4*B*). Overall, we propose that these complex glycosylation sites not only shield the stalk from epitopes by occupying the space around the flexible hinges connecting the neck, HR2, and TM domains but also, contribute to the flexibility of the stalk, which may, in turn, increase the range of motion of the spike head, allowing for more effective sampling of the human cell surface.

On further examination of the glycan–lipid interactions, we observed that the majority of these contacts are formed with POPC or POPE, followed by PSM (*SI Appendix*, Fig. S7). The majority of the interactions between glycans and lipids consist of hydrogen bonds formed between O or N atoms of the *N*-acetyl-D-neuraminic acid or D-galactose sugars present in the glycans and N atoms from PE head group or O atoms in the POPE/POPC phosphate groups. These interactions may further stabilize the inclined conformations of the HR2 domain with respect to the membrane. The contacts with cholesterol are minimal, which is expected as cholesterol is known to insert more deeply in the membrane core compared with the phospholipids (55, 56).

Although because of the unavailability of structural information for unglycosylated spike proteins, no direct evidence for modulation of spike dynamics by glycosylation currently exists, previous studies on other glycoproteins indicate that glycans may play a role in their structure and dynamics. These include mass spectrometry results showing that oligosaccharides in immunoglobulins affect the conformational behavior of their hinge regions (57), where mutations of the glycosylation sites resulted in a change in the rigidity of the interdomain linkers (58). Furthermore, large-scale bending motion of a membrane glycoprotein ectodomain observed by electron microscopy was found to be modulated by the conserved *N*-glycosylations proximal to the joint between its domains (59). It can, therefore, be expected that similar conformational modulatory effects of glycans may be also present in the SARS-CoV-2 spike protein.

### Correlated Interdomain Motions in the Spike.

**Fig. 6.**
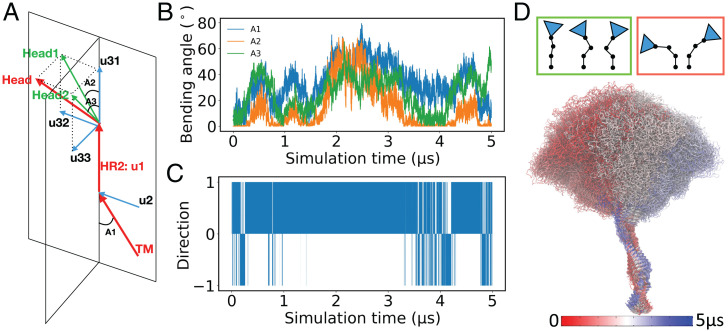
Global dynamics correlation in spike. (*A*) Vectors representing the different domains of the spike (arrows in red): spike head (top arrow), HR2 (middle arrow), TM (bottom arrow), and their orthogonalized subvectors (arrows in blue) in orthogonal planes of spike head (u31, u32, and u33) and TM (u2). Head1 and Head2 (arrows in green) are the portions of the head vector on the HR2–TM plane and the one perpendicular to it, respectively, representing the lateral and axial movements of the head. (*B*) The time series of the A1, A2, and A3 angles introduced in *A* are shown for the fully glycosylated spike. (*C*) The time series of the Gram–Schmidt process for computing the dot product between the u2 and u32 vectors in *A* is shown. A value of +1 means the vectors are pointing in the same direction, and a value of –1 means that they are pointing in the opposite direction. (*D*) The conformational ensemble of the spike generated by taking 100 images evenly distributed over the trajectory of the fully glycosylated spike and superimposing them on the stalk region (the HR2 and TM domains). The color changes from red to blue over the time. The cartoon representations of the spike in *Upper* (the spike head is depicted as triangles, and different stalk domains are connected by lines) in the green box (*Upper Left*) are the favored (highly sampled) conformations, whereas representations in the red box (*Upper Right*) are rarely sampled during the simulations.

The global conformational dynamics of the spike were further examined for the presence of correlated motions between its different domains. A correlation between the motions of the spike head and the TM domain about the HR2 domain is evident in the fully glycosylated spike (Fig. 6 *A* and *B* and Movie S4). A strong Pearson’s coefficient of 0.84 ±0.07 between the Head1 and TM vectors (Fig. 6 *A* and *B*), representing the spike head’s coplanar motion with respect to the HR2–TM domain bending, further confirms the observed correlation in the motions of these domains. This is an interesting observation in that the orientational motions of the different domains of the spike appear to remain interrelated, even though they are connected by flexible hinge regions. Without glycosylations, not only is the magnitude of interdomain motions dampened (*SI Appendix*, Fig. S8), but at the same time, the correlation between them is also reduced, with the nonglycosylated spike showing a relatively lower Pearson’s coefficient, 0.76 ±0.07, for the in-plane motion of the spike head with respect to the HR2–TM domain bending. In general, the correlated motions between the different spike domains can be attributed to its unique tertiary structure and can be considered an intrinsic property of the protein, but at the same time, glycosylation may further amplify these motions through the extensive glycan–lipid and glycan–glycan interactions in the system (Fig. 5). For example, interactions of the glycans located at the bottom end of the HR2 domain with the lipids seem to promote its bending toward the membrane that may, in turn, facilitate the formation of more contacts between the glycans located at the top/opposite end of the HR2 domain and glycans in the neck region, promoting bending of the spike neck and head in the opposite direction.

The Gram–Schmidt analysis (*Materials and Methods*) indicates concerted motions of the spike head and the TM domain about the HR2 domain. Here, the orthonormal vectors representing the spike head and the TM domain are oriented in the same direction about the HR2 domain for 88% of the simulation in the fully glycosylated spike (Fig. 6*C*). The nonglycosylated spike displays a similar behavior, with the orthonormal vectors oriented in the same direction for 89% of the time, further highlighting that these motions are largely a property of the spike structure itself. Topologically, this reflects a configuration where the spike head and the TM domain orient diametrically opposing each other around the HR2 domain, a configuration that may further extend the reach of the spike head and the RBD for the target cellular receptors (Fig. 6*D*). Additionally, considering the fact that the inclination of TM domain in the membrane can be substantial, ranging between 0^∘^ and 30${}^{{}^{\circ}}$ (*SI Appendix*, Fig. S9), the concerted, opposing bending of the spike head and the TM domain may pull the head back to an upward position with respect to the membrane, preventing it from severe inclination and falling into the membrane and thus, maintaining a proper configuration for binding with the ACE2 receptor.

### Role of Palmitoylations in Modulating Membrane Curvature.

**Fig. 7.**
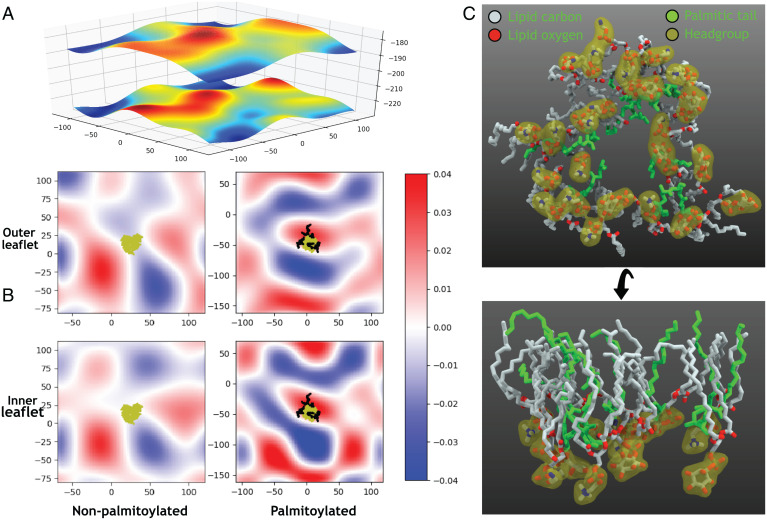
Membrane curvature modulation by palmitoylation. (*A*) Fitted surface (periodic) with lipid head group coordinates used for the calculation of curvature. (*B*) The mean curvature of the fitted membrane surface of the outer and inner leaflets as in *A*. *Left* and *Right* show the curvature profiles at the end of the simulations of nonpalmitoylated and palmitoylated TM domains, respectively. The heavy atoms of the TM domain (yellow) and palmitoyls (black) are also shown. (*C*) Bottom and side views of the membrane lipids proximal to the palmitoylations. The palmitic tails are shown in green, and elements in lipids are colored differently. The lipid head groups are covered in an orange surface. The majority of the interactions between the palmitoyl tails and lipids are hydrophobic, with the palmitoyl tails occupying the space where otherwise, lipid tails would be present.

Protein *S*-palmitoylation, the covalent lipid modification of the Cys residues, widely exists in viral membrane proteins and is reported to contribute to membrane fusion and other processes (33–35). To characterize the effect of spike palmitoylations on the membrane shape, we compared the membrane curvature for the control simulation of nonpalmitoylated spike with the palmoytilated (and fully glycosylated) one, taking into account the periodicity in fitting the lipid head groups. The two simulations share a similar membrane size, but the palmitoylated system seems to deform the membrane more than the nonpalmitoylated system (Fig. 7 *A* and *B*). Keeping in mind that the periodic boundary conditions used in the simulations can suppress long-range curvature effects, comparing the local curvature (in the range of 20 to 50 Å from the center of the TM domain) shows that the palmitoylated TM domain is found within a positively curved membrane, whereas the nonpalmitoylated TM prefers a saddle point. Thus, the palmitoyl tails may induce a positive curvature locally in the vicinity of the TM domain. The observed effect of the palmitoyl tails on the local membrane properties in the vicinity of the protein is complex, primarily and largely dominated by the protein segment they are connected to (Fig. 7*C*). Such tethering effects may promote any pattern of membrane curvature (positive or negative) depending on the protein context.

The lipidation of other viral proteins is relatively well studied. In influenza virus, cryogenic electron tomography (cryo-ET) measurements have shown membrane curvature induced by hemagglutinin (HA) palmitoylation (34). Apart from this, nonacylated HA mutants of influenza virus show a severe negative effect on the membrane fusion step of the infection process (60). As shown by the results above, SARS-CoV-2 spike palmitoylation may similarly contribute to the bending of the membrane, especially locally, and thus, may play a role in the viral fusion with the host cell membrane.

## Conclusions

**Fig. 8.**
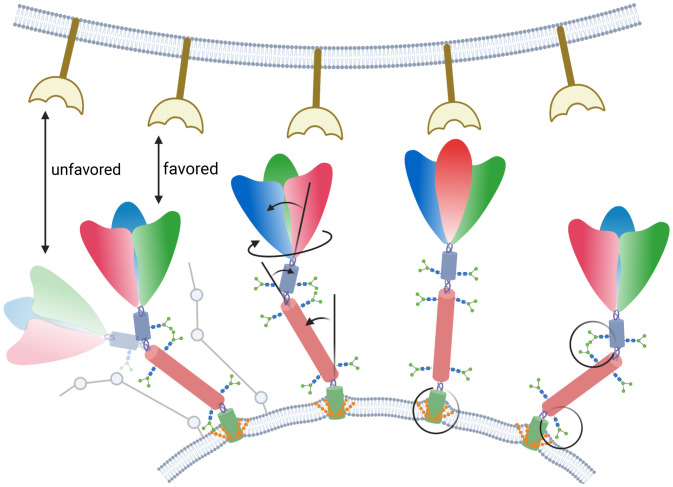
Impact of posttranslational modifications on spike global dynamics and membrane deformation. The host cellular membrane with binding receptors is shown in *Upper*, and the viral envelope containing multiple spikes is shown in *Lower*. Each spike head is shown in three colors, representing the three constituting monomers, and the different stalk domains are also shown in different colors. Spike copies shown from left to right highlight the following results: 1) correlated motions of the spike head and stalk, allowing reorientation of the spike head with respect to the receptor (stick representations of the spike in favorable and unfavorable orientations are also shown on the side); 2) global motion of the spike head around the stalk (twisting and bending motions), allowing it to broaden its reach for the cellular receptors and optimally binding to it; 3) membrane curvature induced by palmitoylation potentially plays a role in viral fusion; and 4) glycan–lipid and glycan–glycan interactions restricting/modulating the stalk motions.

The spike protein of SARS-CoV-2 constitutes a major component in the viral infection and therefore, the main target for both diagnostics and vaccines developed for the disease caused by the virus. To better understand the many mechanistic steps the spike is involved in, it is imperative to characterize its structure and dynamics in the most detailed manner and under the most realistic conditions possible. We report here a full model for the full-length spike in its native, glycosylated, palmitoylated, and membrane-bound form, which we use for several microseconds of atomistic simulations highlighting some of the dynamics employed by the spike to search for and locate the host cell receptors effectively (Fig. 8). The hinge regions identified in the stalk domain directly modulate the conformational landscape of the spike head, where the RBDs reside, and may thus represent important structural elements regulating the effective role of the spike in binding its receptor. These highly conserved hinges may offer targets for developing alternative antibodies and therapeutics that bind them and modulate their structure/dynamics and therefore, the effectiveness of the spike protein. In addition, we propose possible roles for posttranslational modifications of spike, specifically glycosylation and palmitoylation, in modulating its dynamics. The results provide a deeper understanding of the intricate roles and functions of the different elements of the spike, each evolved in order to maximize the chance of successful infection and transmission of the genetic material to the host cell. Extended simulations studying the spike behavior in a full viral envelope, as recently done for the SARS-CoV-2 Delta variant (61), will allow further characterization of the impact of a more crowded environment of the envelope and its proteins in modulating the structure and dynamics of the spike and will provide additional insight into the inner working of the virus.

## Materials and Methods

An extended version of the methods is available as *SI Appendix*.

As a first step, a full-length spike was constructed using recent cryo-EM structures of the spike head from SARS-CoV-2 [Protein Data Bank (PDB) ID codes 6VYB and 6VXX (9)], SARS-CoV [PDB ID codes 5X58 (62), 5XLR (63), and 6CRW (64)], and MERS-CoV [PDB ID code 6Q04 (65)] along with the SARS-CoV-2 RBD structure in complex with human receptor ACE2 [PDB ID code 6M17 (66)], to which missing protein segments (e.g., the fusion peptide) (16, 17) were added in MOE (67) using either template- or fragment-based modeling. Missing regions without a suitable template were constructed using the Robetta protein structure prediction server (68, 69). The spike neck was also modeled using Robetta, and the HR2 domain was homology modeled using the structure of SARS-CoV protein [PDB ID code 2FXP (42)]. The modeled regions were tested against secondary structure predictions by JPred4 (70).

The TM domain, which is important for spike stabilization (19, 71), shows low homology to the available HIV virus (30.4% identity), making it unsuitable for template-based modeling (43). After prediction of the TM region from the sequence in Transmembrane Helices; Hidden Markov Model (TMHMM) (72), a TM monomer was constructed and embedded in a membrane with the lipid composition of ERGIC (22, 73), where coronaviruses assemble (23) (*SI Appendix*, Table S1). After 100 ns of relaxation of the monomer in the membrane, multiple trimeric configurations were generated using Multimer Docking in ClusPro (74). The two best trimeric TM configurations were embedded in the membrane, solvated, and equilibrated for 200 ns each. The stability of these models was evaluated by calculating rmsd, TM tilt/inclination in the membrane, and intermonomeric coordination number [coordNum in the collective variables (COLVARS) module (75, 76) of Visual Molecular Dynamics (VMD) (77)]. The most stable trimeric conformation was used to construct the full-length spike structure. The structure for the luminal C-terminal region was modeled with Robetta. The structure of the full-length spike was then constructed by assembling the experimental spike head and the individually constructed domains. Missing loops were then filled with MODELER (78). Based on mass spectrometry data (38, 39), we then added a total of 22 *N*-glycans and 1 *O*-glycan (39) to each spike monomer (*SI Appendix*, Table S2) using Glycan Reader & Modeler (30, 79) in CHARMM-GUI (80, 81). Palmitoylations at the endodomain (37) were modeled based on SARS-CoV data (37) (residues 1,236, 1,240, and 1,241).

MD simulations of the full-length, membrane-embedded spike structure in both glycosylated and nonglycosylated forms (5 µs each) were then performed in NAMD (82, 83) using CHARMM36m (84, 85). The simulations were performed as an NPT ensemble (310 K and 1 bar) employing Langevin thermostat and barostat (86, 87) with the particle mesh Ewald method for electrostatic forces (88) and applying SHAKE to constrain bonds with hydrogen atoms (89).

The global dynamics (*SI Appendix*, Fig. S10) of the protein were characterized in terms of the positional and orientational behavior of the spike head with respect to the different stalk domains. The potential effect of glycosylation on these motions was analyzed in relation to their direct interactions with the lipid bilayer as well as contacts between the glycans at the interfaces of different domains. Since the palmitoylation tails contribute to membrane deformation (34), we also quantified the potential effect of palmitoylation on the membrane curvature. Multiple sequence alignment was carried out using the Multiple Alignment using Fast Fourier Transform (MAFFT) program (90) and visualized using Jalview (91). The solvent accessible surface area was calculated with the Shrake–Rupley algorithm (92).

## Supplementary Material

Supplementary File

Supplementary File

Supplementary File

Supplementary File

Supplementary File

## Data Availability

The starting structures for both the glycosylated and nonglycolated spike simulation systems along with the trajectories have been deposited in the publicly accessible database Galaxy (https://usegalaxy.org/u/tianle_chen/h/spiketrajectory) (93).

## References

[1] C. Huang ., Clinical features of patients infected with 2019 novel coronavirus in Wuhan, China. Lancet 395, 497–506 (2020). 31986264 10.1016/S0140-6736(20)30183-5 PMC7159299

[2] J. F. W. Chan ., A familial cluster of pneumonia associated with the 2019 novel coronavirus indicating person-to-person transmission: A study of a family cluster. Lancet 395, 514–523 (2020). 31986261 10.1016/S0140-6736(20)30154-9 PMC7159286

[3] M. Chan-Yeung, R. H. Xu, SARS: Epidemiology. *Respirology* **8** (suppl.), S9–S14 (2003). 10.1046/j.1440-1843.2003.00518.x PMC7169193 15018127

[4] A. Zumla, D. S. Hui, S. Perlman, Middle East respiratory syndrome. Lancet 386, 995–1007 (2015). 26049252 10.1016/S0140-6736(15)60454-8 PMC4721578

[5] W. Li ., Angiotensin-converting enzyme 2 is a functional receptor for the SARS coronavirus. Nature 426, 450–454 (2003). 14647384 10.1038/nature02145 PMC7095016

[6] Y. Wan, J. Shang, R. Graham, R. S. Baric, F. Li, Receptor recognition by the novel coronavirus from Wuhan: An analysis based on decade-long structural studies of SARS coronavirus. J. Virol. 94, e00127-20 (2020). 31996437 10.1128/JVI.00127-20 PMC7081895

[7] T. Tang, M. Bidon, J. A. Jaimes, G. R. Whittaker, S. Daniel, Coronavirus membrane fusion mechanism offers a potential target for antiviral development. Antiviral Res. 178, 104792 (2020). 32272173 10.1016/j.antiviral.2020.104792 PMC7194977

[8] D. Wrapp ., Cryo-EM structure of the 2019-nCoV spike in the prefusion conformation. Science 367, 1260–1263 (2020). 32075877 10.1126/science.abb2507 PMC7164637

[9] A. C. Walls ., Structure, function, and antigenicity of the SARS-CoV-2 spike glycoprotein. Cell 181, 281–292.e6 (2020). 32155444 10.1016/j.cell.2020.02.058 PMC7102599

[10] M. A. Tortorici ., Ultrapotent human antibodies protect against SARS-CoV-2 challenge via multiple mechanisms. Science 370, 950–957 (2020). 32972994 10.1126/science.abe3354 PMC7857395

[11] R. Henderson ., Controlling the SARS-CoV-2 spike glycoprotein conformation. Nat. Struct. Mol. Biol. 27, 925–933 (2020). 32699321 10.1038/s41594-020-0479-4 PMC8581954

[12] Z. Ke ., Structures and distributions of SARS-CoV-2 spike proteins on intact virions. Nature 588, 498–502 (2020). 32805734 10.1038/s41586-020-2665-2 PMC7116492

[13] C. Toelzer ., Free fatty acid binding pocket in the locked structure of SARS-CoV-2 spike protein. Science 370, 725–730 (2020). 32958580 10.1126/science.abd3255 PMC8050947

[14] J. Juraszek ., Stabilizing the closed SARS-CoV-2 spike trimer. Nat. Commun. 12, 244 (2021). 33431842 10.1038/s41467-020-20321-x PMC7801441

[15] Y. Cai ., Distinct conformational states of SARS-CoV-2 spike protein. Science 369, 1586–1592 (2020). 32694201 10.1126/science.abd4251 PMC7464562

[16] L. K. Tamm, X. Han, Viral fusion peptides: A tool set to disrupt and connect biological membranes. Biosci. Rep. 20, 501–518 (2000). 11426691 10.1023/a:1010406920417

[17] J. M. White, S. E. Delos, M. Brecher, K. Schornberg, Structures and mechanisms of viral membrane fusion proteins: Multiple variations on a common theme. Crit. Rev. Biochem. Mol. Biol. 43, 189–219 (2008). 18568847 10.1080/10409230802058320 PMC2649671

[18] A. C. Walls ., Tectonic conformational changes of a coronavirus spike glycoprotein promote membrane fusion. Proc. Natl. Acad. Sci. U.S.A. 114, 11157–11162 (2017). 29073020 10.1073/pnas.1708727114 PMC5651768

[19] R. Broer, B. Boson, W. Spaan, F. L. Cosset, J. Corver, Important role for the transmembrane domain of severe acute respiratory syndrome coronavirus spike protein during entry. J. Virol. 80, 1302–1310 (2006). 16415007 10.1128/JVI.80.3.1302-1310.2006 PMC1346921

[20] E. B. Thorp, J. A. Boscarino, H. L. Logan, J. T. Goletz, T. M. Gallagher, Palmitoylations on murine coronavirus spike proteins are essential for virion assembly and infectivity. J. Virol. 80, 1280–1289 (2006). 16415005 10.1128/JVI.80.3.1280-1289.2006 PMC1346925

[21] Y. Watanabe, T. A. Bowden, I. A. Wilson, M. Crispin, Exploitation of glycosylation in enveloped virus pathobiology. Biochim. Biophys. Acta, Gen. Subj. 1863, 1480–1497 (2019). 31121217 10.1016/j.bbagen.2019.05.012 PMC6686077

[22] A. Schweizer, H. Clausen, G. van Meer, H. P. Hauri, Localization of O-glycan initiation, sphingomyelin synthesis, and glucosylceramide synthesis in Vero cells with respect to the endoplasmic reticulum-Golgi intermediate compartment. J. Biol. Chem. 269, 4035–4041 (1994). 8307959

[23] B. G. Hogue, C. E. Machamer, Coronavirus Structural Proteins and Virus Assembly in Nidoviruses (American Society of Microbiology, 2008), pp. 179–200.

[24] O. C. Grant, D. Montgomery, K. Ito, R. J. Woods, Analysis of the SARS-CoV-2 spike protein glycan shield reveals implications for immune recognition. Sci. Rep. 10, 14991 (2020). 32929138 10.1038/s41598-020-71748-7 PMC7490396

[25] S. A. Serapian ., The answer lies in the energy: How simple atomistic molecular dynamics simulations may hold the key to epitope prediction on the fully glycosylated SARS-CoV-2 spike protein. J. Phys. Chem. Lett. 11, 8084–8093 (2020). 32885971 10.1021/acs.jpclett.0c02341 PMC7491317

[26] M. Gur ., Conformational transition of SARS-CoV-2 spike glycoprotein between its closed and open states. J. Chem. Phys. 153, 075101 (2020). 32828084 10.1063/5.0011141

[27] L. Fallon ., Free energy landscapes from SARS-CoV-2 spike glycoprotein simulations suggest that RBD opening can be modulated via interactions in an allosteric pocket. J. Am. Chem. Soc. 143, 11349–11360 (2021). 34270232 10.1021/jacs.1c00556 PMC8315243

[28] M. I. Zimmerman ., SARS-CoV-2 simulations go exascale to predict dramatic spike opening and cryptic pockets across the proteome. Nat. Chem. 13, 651–659 (2021). 34031561 10.1038/s41557-021-00707-0 PMC8249329

[29] T. Sztain ., A glycan gate controls opening of the SARS-CoV-2 spike protein. Nat. Chem. 13, 963–968 (2021). 34413500 10.1038/s41557-021-00758-3 PMC8488004

[30] H. Woo ., Developing a fully glycosylated full-length SARS-CoV-2 spike protein model in a viral membrane. J. Phys. Chem. B 124, 7128–7137 (2020). 32559081 10.1021/acs.jpcb.0c04553 PMC7341691

[31] L. Casalino ., Beyond shielding: The roles of glycans in the SARS-CoV-2 spike protein. ACS Cent. Sci. 6, 1722–1734 (2020). 33140034 10.1021/acscentsci.0c01056 PMC7523240

[32] M. Sikora ., Computational epitope map of SARS-CoV-2 spike protein. PLOS Comput. Biol. 17, e1008790 (2021). 33793546 10.1371/journal.pcbi.1008790 PMC8016105

[33] C. Aicart-Ramos, R. A. Valero, I. Rodriguez-Crespo, Protein palmitoylation and subcellular trafficking. Biochim. Biophys. Acta 1808, 2981–2994 (2011). 21819967 10.1016/j.bbamem.2011.07.009

[34] P. Chlanda ., Palmitoylation contributes to membrane curvature in influenza A virus assembly and hemagglutinin-mediated membrane fusion. J. Virol. 91, e00947-17 (2017). 28794042 10.1128/JVI.00947-17 PMC5640829

[35] D. L. Parton, A. Tek, M. Baaden, M. S. P. Sansom, Formation of raft-like assemblies within clusters of influenza hemagglutinin observed by MD simulations. PLOS Comput. Biol. 9, e1003034 (2013). 23592976 10.1371/journal.pcbi.1003034 PMC3623702

[36] A. M. Ernst ., S-Palmitoylation sorts membrane cargo for anterograde transport in the Golgi. Dev. Cell 47, 479–493.e7 (2018). 30458139 10.1016/j.devcel.2018.10.024 PMC6251505

[37] C. M. Petit ., Palmitoylation of the cysteine-rich endodomain of the SARS-coronavirus spike glycoprotein is important for spike-mediated cell fusion. Virology 360, 264–274 (2007). 17134730 10.1016/j.virol.2006.10.034 PMC7103323

[38] A. Shajahan, N. T. Supekar, A. S. Gleinich, P. Azadi, Deducing the N- and O-glycosylation profile of the spike protein of novel coronavirus SARS-CoV-2. Glycobiology 30, 981–988 (2020). 32363391 10.1093/glycob/cwaa042 PMC7239183

[39] Y. Watanabe, J. D. Allen, D. Wrapp, J. S. McLellan, M. Crispin, Site-specific glycan analysis of the SARS-CoV-2 spike. Science 369, 330–333 (2020). 32366695 10.1126/science.abb9983 PMC7199903

[40] W. Xu, M. Wang, D. Yu, X. Zhang, Variations in SARS-CoV-2 spike protein cell epitopes and glycosylation profiles during global transmission course of COVID-19. Front. Immunol. 11, 565278 (2020). 33013929 10.3389/fimmu.2020.565278 PMC7509417

[41] B. Turoňová ., In situ structural analysis of SARS-CoV-2 spike reveals flexibility mediated by three hinges. Science 370, 203–208 (2020). 32817270 10.1126/science.abd5223 PMC7665311

[42] S. Hakansson-McReynolds, S. Jiang, L. Rong, M. Caffrey, Solution structure of the severe acute respiratory syndrome-coronavirus heptad repeat 2 domain in the prefusion state. J. Biol. Chem. 281, 11965–11971 (2006). 16507566 10.1074/jbc.M601174200 PMC8099417

[43] J. Dev ., Structural basis for membrane anchoring of HIV-1 envelope spike. Science 353, 172–175 (2016). 27338706 10.1126/science.aaf7066 PMC5085267

[44] M. G. Teese, D. Langosch, Role of GxxxG motifs in transmembrane domain interactions. Biochemistry 54, 5125–5135 (2015). 26244771 10.1021/acs.biochem.5b00495

[45] H. Yao ., Molecular architecture of the SARS-CoV-2 virus. Cell 183, 730–738.e13 (2020). 32979942 10.1016/j.cell.2020.09.018 PMC7474903

[46] Y. K. Choi ., Structure, dynamics, receptor binding, and antibody binding of the fully glycosylated full-length sars-cov-2 spike protein in a viral membrane. J. Chem. Theory Comput. 17, 2479–2487 (2021). 33689337 10.1021/acs.jctc.0c01144 PMC8047829

[47] M. J. Betts, R. B. Russell, “Amino acid properties and consequences of substitutions” in Bioinformatics for Geneticists, M. R. Barnes, I. Gray, Eds. (Wiley, New York, NY, 2003), **vol**. 317, pp. 289–316.

[48] B. K. Ho, E. A. Coutsias, C. Seok, K. A. Dill, The flexibility in the proline ring couples to the protein backbone. Protein Sci. 14, 1011–1018 (2005). 15772308 10.1110/ps.041156905 PMC2253451

[49] K. W. Ng ., Preexisting and de novo humoral immunity to SARS-CoV-2 in humans. Science 370, 1339–1343 (2020). 33159009 10.1126/science.abe1107 PMC7857411

[50] P. Nguyen-Contant ., S protein-reactive IgG and memory B cell production after human SARS-CoV-2 infection includes broad reactivity to the S2 subunit. MBio 11, e01991-20 (2020). 32978311 10.1128/mBio.01991-20 PMC7520599

[51] P. Pandey ., Targeting SARS-CoV-2 spike protein of COVID-19 with naturally occurring phytochemicals: An in silico study for drug development. J. Biomol. Struct. Dyn. 39, 6306–6316 (2021). 32698689 10.1080/07391102.2020.1796811 PMC7441770

[52] J. Te Riet, B. Joosten, I. Reinieren-Beeren, C. G. Figdor, A. Cambi, N-glycan mediated adhesion strengthening during pathogen-receptor binding revealed by cell-cell force spectroscopy. Sci. Rep. 7, 6713 (2017). 28751750 10.1038/s41598-017-07220-w PMC5532264

[53] S. J. Yoon ., Interaction of N-linked glycans, having multivalent GlcNAc termini, with GM3 ganglioside. Glycoconj. J. 23, 639–649 (2006). 17115280 10.1007/s10719-006-9001-4

[54] S. J. Yoon ., Self-recognition of N-linked glycans with multivalent GlcNAc, determined as ceramide mimetic conjugate. Glycobiology 17, 1007–1014 (2007). 17609198 10.1093/glycob/cwm069

[55] D. Marquardt ., Lipid bilayer thickness determines cholesterol’s location in model membranes. Soft Matter 12, 9417–9428 (2016). 27801465 10.1039/c6sm01777k

[56] J. Dai, M. Alwarawrah, J. Huang, Instability of cholesterol clusters in lipid bilayers and the cholesterol’s Umbrella effect. J. Phys. Chem. B 114, 840–848 (2010). 20041657 10.1021/jp909061h PMC2818971

[57] R. Plomp ., Hinge-region O-glycosylation of human immunoglobulin G3 (IgG3). Mol. Cell. Proteomics 14, 1373–1384 (2015). 25759508 10.1074/mcp.M114.047381 PMC4424406

[58] J. S. Klein, S. Jiang, R. P. Galimidi, J. R. Keeffe, P. J. Bjorkman, Design and characterization of structured protein linkers with differing flexibilities. Protein Eng. Des. Sel. 27, 325–330 (2014). 25301959 10.1093/protein/gzu043 PMC4191447

[59] K. Matoba ., Conformational freedom of the LRP6 ectodomain is regulated by N-glycosylation and the binding of the wnt antagonist Dkk1. Cell Rep. 18, 32–40 (2017). 28052259 10.1016/j.celrep.2016.12.017

[60] T. Sakai, R. Ohuchi, M. Ohuchi, Fatty acids on the A/USSR/77 influenza virus hemagglutinin facilitate the transition from hemifusion to fusion pore formation. J. Virol. 76, 4603–4611 (2002). 11932425 10.1128/JVI.76.9.4603-4611.2002 PMC155084

[61] A. Dommer ., #COVIDisAirborne: AI-enabled multiscale computational microscopy of delta SARS-CoV-2 in a respiratory aerosol. bioRxiv [Preprint] (2021). 10.1101/2021.11.12.468428 (Published 15 Nov. 2021). PMC9527558 36647365

[62] Y. Yuan ., Cryo-EM structures of MERS-CoV and SARS-CoV spike glycoproteins reveal the dynamic receptor binding domains. Nat. Commun. 8, 15092 (2017). 28393837 10.1038/ncomms15092 PMC5394239

[63] M. Gui ., Cryo-electron microscopy structures of the SARS-CoV spike glycoprotein reveal a prerequisite conformational state for receptor binding. Cell Res. 27, 119–129 (2017). 28008928 10.1038/cr.2016.152 PMC5223232

[64] R. N. Kirchdoerfer ., Stabilized coronavirus spikes are resistant to conformational changes induced by receptor recognition or proteolysis. Sci. Rep. 8, 15701 (2018). 30356097 10.1038/s41598-018-34171-7 PMC6200764

[65] Y. J. Park ., Structures of MERS-CoV spike glycoprotein in complex with sialoside attachment receptors. Nat. Struct. Mol. Biol. 26, 1151–1157 (2019). 31792450 10.1038/s41594-019-0334-7 PMC7097669

[66] R. Yan ., Structural basis for the recognition of SARS-CoV-2 by full-length human ACE2. Science 367, 1444–1448 (2020). 32132184 10.1126/science.abb2762 PMC7164635

[67] S. Vilar, G. Cozza, S. Moro, Medicinal chemistry and the molecular operating environment (MOE): Application of QSAR and molecular docking to drug discovery. Curr. Top. Med. Chem. 8, 1555–1572 (2008). 19075767 10.2174/156802608786786624

[68] S. Raman ., Structure prediction for CASP8 with all-atom refinement using Rosetta. Proteins 77 (suppl. 9), 89–99 (2009). 19701941 10.1002/prot.22540 PMC3688471

[69] Y. Song ., High-resolution comparative modeling with RosettaCM. Structure 21, 1735–1742 (2013). 24035711 10.1016/j.str.2013.08.005 PMC3811137

[70] A. Drozdetskiy, C. Cole, J. Procter, G. J. Barton, JPred4: A protein secondary structure prediction server. Nucleic Acids Res. 43 (W1), W389-94 (2015). 25883141 10.1093/nar/gkv332 PMC4489285

[71] B. J. Bosch, C. A. de Haan, S. L. Smits, P. J. Rottier, Spike protein assembly into the coronavirion: Exploring the limits of its sequence requirements. Virology 334, 306–318 (2005). 15780881 10.1016/j.virol.2005.02.001 PMC7111810

[72] A. Krogh, B. Larsson, G. von Heijne, E. L. Sonnhammer, Predicting transmembrane protein topology with a hidden Markov model: Application to complete genomes. J. Mol. Biol. 305, 567–580 (2001). 11152613 10.1006/jmbi.2000.4315

[73] J. Krijnse-Locker, M. Ericsson, P. J. Rottier, G. Griffiths, Characterization of the budding compartment of mouse hepatitis virus: Evidence that transport from the RER to the Golgi complex requires only one vesicular transport step. J. Cell Biol. 124, 55–70 (1994). 8294506 10.1083/jcb.124.1.55 PMC2119890

[74] D. Kozakov ., The ClusPro web server for protein-protein docking. Nat. Protoc. 12, 255–278 (2017). 28079879 10.1038/nprot.2016.169 PMC5540229

[75] G. Fiorin, M. L. Klein, J. Hénin, Using collective variables to drive molecular dynamics simulations. Mol. Phys. 111, 3345–3362 (2013).

[76] E. A. Coutsias, C. Seok, K. A. Dill, Using quaternions to calculate RMSD. J. Comput. Chem. 25, 1849–1857 (2004). 15376254 10.1002/jcc.20110

[77] W. Humphrey, A. Dalke, K. Schulten, VMD: Visual molecular dynamics. J. Mol. Graph. 14, 33–38 (1996). 8744570 10.1016/0263-7855(96)00018-5

[78] A. Fiser, R. K. G. Do, A. Šali, Modeling of loops in protein structures. Protein Sci. 9, 1753–1773 (2000). 11045621 10.1110/ps.9.9.1753 PMC2144714

[79] S. J. Park ., CHARMM-GUI Glycan Modeler for modeling and simulation of carbohydrates and glycoconjugates. Glycobiology 29, 320–331 (2019). 30689864 10.1093/glycob/cwz003 PMC6422236

[80] S. Jo, T. Kim, V. G. Iyer, W. Im, CHARMM-GUI: A web-based graphical user interface for CHARMM. J. Comput. Chem. 29, 1859–1865 (2008). 18351591 10.1002/jcc.20945

[81] E. L. Wu ., CHARMM-GUI Membrane Builder toward realistic biological membrane simulations. J. Comput. Chem. 35, 1997–2004 (2014). 25130509 10.1002/jcc.23702 PMC4165794

[82] J. C. Phillips ., Scalable molecular dynamics with NAMD. J. Comput. Chem. 26, 1781–1802 (2005). 16222654 10.1002/jcc.20289 PMC2486339

[83] J. C. Phillips ., Scalable molecular dynamics on CPU and GPU architectures with NAMD. J. Chem. Phys. 153, 044130 (2020). 32752662 10.1063/5.0014475 PMC7395834

[84] K. Hart ., Optimization of the CHARMM additive force field for DNA: Improved treatment of the BI/BII conformational equilibrium. J. Chem. Theory Comput. 8, 348–362 (2012). 22368531 10.1021/ct200723y PMC3285246

[85] J. B. Klauda ., Update of the CHARMM all-atom additive force field for lipids: Validation on six lipid types. J. Phys. Chem. B 114, 7830–7843 (2010). 20496934 10.1021/jp101759q PMC2922408

[86] G. J. Martyna, D. J. Tobias, M. L. Klein, Constant pressure molecular dynamics algorithms. J. Chem. Phys. 101, 4177–4189 (1994).

[87] S. E. Feller, Y. Zhang, R. W. Pastor, Constant pressure molecular dynamics simulation: The Langevin piston method. J. Chem. Phys. 103, 4613–4621 (1995).

[88] T. Darden, D. York, L. Pedersen, Particle mesh Ewald: An N·log(N) method for Ewald sums in large systems. J. Chem. Phys. 98, 10089–10092 (1993).

[89] J. P. Ryckaert, G. Ciccotti, H. J. C. Berendsen, Numerical integration of the Cartesian equations of motion of a system with constraints: Molecular dynamics of *n*-alkanes. J. Comput. Phys. 23, 327–341 (1977).

[90] K. Katoh, K. Misawa, K. Kuma, T. Miyata, MAFFT: A novel method for rapid multiple sequence alignment based on fast Fourier transform. Nucleic Acids Res. 30, 3059–3066 (2002). 12136088 10.1093/nar/gkf436 PMC135756

[91] M. Clamp, J. Cuff, S. M. Searle, G. J. Barton, The Jalview Java alignment editor. Bioinformatics 20, 426–427 (2004). 14960472 10.1093/bioinformatics/btg430

[92] A. Shrake, J. A. Rupley, Environment and exposure to solvent of protein atoms. Lysozyme and insulin. J. Mol. Biol. 79, 351–371 (1973). 4760134 10.1016/0022-2836(73)90011-9

[93] T. Chen, SARS-CoV2 spike simulation trajectory. Galaxy Data Repository. https://usegalaxy.org/u/tianle_chen/h/spiketrajectory. Deposited 20 February 2022.

