# Supplementary material for: Posttranslational modifications optimize the ability of SARS-CoV-2 spike for effective interaction with host cell receptors
Source: Proc Natl Acad Sci U S A. 2022 Jun 23;119(28):e2119761119. doi: 10.1073/pnas.2119761119 (PMC9282386; doi:10.1073/pnas.2119761119)
Supplement: Supplementary File [file pnas.2119761119.sapp.pdf]

PNAS
www.pnas.org

Supplementary Information for

Post-Translational Modifications Optimize the Ability of SARS-CoV-2 Spike for Effective Interaction with Host Cell Receptors

Karan Kapoor, Tianle Chen and Emad Tajkhorshid

Emad Tajkhorshid
E-mail: emad@illinois.edu

This PDF file includes:

Supplementary text
Figs. S1 to S10
Tables S1 to S3
Legends for Movies S1 to S4
SI References

Other supplementary materials for this manuscript include the following:

Movies S1 to S4

Supporting Information Text

Extended Methods

Modeling of full-length spike. Topologically, the spike can be divided into two main regions - the spike head, which includes the RBD that binds to the host cell receptor, and an extended stalk consisting of a short spike neck, the HR2 domain, and the TM domain tethering the spike to the viral envelope. In addition, the spike also contains a cysteine-rich, disordered C-terminal region inside the envelope. Since the experimental structures have only been resolved for the spike head, as the first step, we model the missing regions to construct a full-length spike structure, which is then palmitoylated at the TM/endodomain and fully-glycosylated, before performing MD simulations. The different steps involved in this process are described below.

Constructing missing regions of the spike head. The recent cryo-EM structures of the spike head don't provide coordinates for a number of missing regions including a short peptide segment called the fusion peptide, known to to be important for membrane binding and insertion (1, 2), as well as a number of loops. These missing regions were generated using either template- or fragment-based modeling as described below. Firstly, the cryo-EM structures of the spike head from SARS-CoV-2 (PDB: 6VYB and 6VXX (3)), SARS-CoV (PDB: 5X58 (4), 5XLR (5), 6CRW (6)) and MERS-CoV (PDB: 6Q04 (7)), along with the SARS-CoV-2 RBD structure in complex with human receptor ACE2 (PDB: 6M17 (8)) were aligned and using sequence- and structure-based alignment in MOE (9). The missing regions were then constructed in MOE by grafting a suitable template region from the aligned structures and using local superimposition based on the flanking residues of the template region. Residues in the grafted templates from other CoV structures were mutated back to those in the SARS-CoV-2 sequence. Missing loop regions in the spike head without a suitable template were constructed using Rosetta ab-initio fragment assembly in the Robetta protein structure prediction server (10, 11).

Modeling the neck and the HR2 domain. Due to lack of a suitable template, the spike neck was modeled using ab-initio modeling in Robetta. The HR2 domain was homology-modeled using the NMR-resolved structure of homologous SARS-CoV (PDB: 2FXP (12)) as the template in MOE. These modeled regions were validated against secondary structure predictions by JPred4 (13).

Developing a trimeric TM model. The structural information about the TM region of SARS-CoV-2 spike, which is highly conserved among coronaviruses and known to play a role in the spike assembly and stabilization (14, 15), is limited, as no structure for this domain has been resolved. The available structure from the HIV virus displays a low sequence homology (30.4 % identity), making it unsuitable for template-based modeling (16). The TM domain of SARS-CoV-2 was thus built in an ab initio manner. Firstly, the location of the TM region in the spike sequence was predicted using TMHMM (17), a bioinformatics-based approach for predicting membrane spanning regions in protein sequences. Using the predicted TM sequence, a TM helix monomer was constructed in MOE. The monomer was then placed in a membrane patch using CHARMM-GUI (18, 19) with a lipid composition based on the lipoprofiles of the ERGIC (20, 21), where coronaviruses assemble (22). The specific lipid compositions of the two leaflets are provided in Table S1. After solvating the system, 1,000 minimization steps and 100 ns equilibration were performed under NPT conditions to relax the monomeric structure in the membrane. Next, in order to construct the trimeric assembly of the TM domain, multiple trimeric configurations of the TM helix were generated using `Multimer Docking` in ClusPro (23), a fast Fourier transform-based, rigid-body docking method. Briefly, a total of 10^9 docking models were generated out of which a subset of 10^3 models were selected based on the predicted binding-energy score of Cluspro. We then clustered these models using RMSD and ranked them according to their populations. The models in the top 10 clusters were visually inspected for the expected symmetry between the individual TM monomers and their membrane orientations, and the two best trimeric TM configurations were selected for additional refinement in MD simulations.

The two selected trimeric TM configurations were similarly embedded in the membrane, solvated, energy-minimized, and equilibrated for 200 ns each. The stability of these models was evaluated by calculating the following metrics: i) RMSD of the TM domain with respect to the starting structure using C_α atoms, ii) TM tilt/inclination, which was measured as the angle between the third principal axis of the moment of inertia of the heavy atoms in each TM monomer and that of the whole TM trimer, and iii) coordination number (`coordNum` implemented in the collective variables (COLVARS) module (24, 25) of VMD (26)) quantifying the number of heavy atom pairs within a cutoff distance, between each TM monomer and the other two TM monomers, using a half-contact value of d_0=4.5 Å. Here, for $d \ll d_0$ the contact value is close to 1, at $d = d_0$ the value is 0.5, and at $d \gg d_0$ the value $\sim$0. The total contact is then calculated by summing over all heavy-atom pairs in the two groups. The most stable TM trimeric conformation thus identified using the above three structural metrics was then used to construct the full-length spike structure.

Constructing the C-terminal region. The C-terminal region, located in the lumen of the viral envelope, is predicted to be intrinsically disordered, through secondary structure predictions by JPred4, and does not have a suitable template structure. We used Robetta to generate multiple ab intio models of this region. From these, a model was selected based on its spatial arrangement with respect to the TM trimeric domain, namely, a model without any steric clashes when connected to the membrane-inserted TM trimer.

Assembling the full-length spike. The structure of the full-length spike was constructed by assembling together the spike head and the individually constructed domains of the stalk region. The structures of the individual domains were aligned using VMD, with the sequential gaps between them ranging between 3-5 residues, which were filled using loop modeling in MODELLER (27).

Spike glycosylation and palmitoylations. The surface of the SARS-CoV-2 spike is known to be extensively glycosylated (28), but structural information on this feature is by and large missing from the available structures. Recent mass spectrometry studies have successfully identified both the spike's glycosylation sites as well as their specific chemical compositions (28, 29). Utilizing this information, we added a total of 22 N-glycans and 1 O-glycan to each of the spike monomers (Table S2).

SARS-CoV-2 spike is also known to be palmitoylated at the endodomain (30), but its specific palmitoylation residues have not been determined. Since the cysteine-rich endodomain of the SARS-CoV spike shares ∼95% sequence identity with the SARS-CoV-2 spike, we used the palmitoylation data from the mutagenesis analysis of the cysteine clusters in the former (31) as a reference for palmoytilation of the SARS-CoV-2 spike. Thus, we adopted 3 palmitoylation sites (residues 1,236, 1,240 and 1,241) in the first two cysteine-rich clusters of the C-terminal region in each spike monomer and covalently linked palmitoyl groups to them.

MD simulations of membrane-bound spike. In order to probe the conformational dynamics of the entire spike as well as the motions displayed by the individual domains, we carried out MD simulations of the full-length, membrane-embedded spike structure, both in glycosylated and non-glycosylated forms. The following sections provide more details on the simulation system construction and the MD protocol used.

System preparation. The final membrane-embedded, full-length spike system was prepared with the CHARMM-GUI webserver (18, 19). 22 N-glycosylations and 1 O-glycosylation were added to each spike monomer using `Glycan Reader & Modeler` in CHARMM-GUI (32, 33). Three palmitic acid tails were also added to cysteine residues 1,236, 1,240 and 1,241 in the endodomain of each monomer. A total of 15 disulfide bonds were introduced between specific cysteine pairs (15-136, 131-166, 291-301, 336-361, 379-432, 391-525, 480-488, 538-590, 617-649, 662-671, 738-760, 743-749, 840-851, 1,032-1,043 and 1,082-1,126). Following this, the spike protein was inserted into a lipid bilayer with ERGIC composition described above (20, 21). The system was then solvated with water and ionized with 0.15 M NaCl, resulting in a simulation system with approximate dimensions of $250 \times 250 \times 390\,\text{Å}^3$ with ~ 2.3 million atoms.

MD simulations. MD simulations were performed using NAMD2, a highly scalable MD engine (34, 35). The CHARMM36m force field was used to represent the proteins, lipids, glycosylations, palmitoylations, and ions (36, 37). The simulations were performed as an NPT ensemble with the temperature and pressure maintained at 310 K and 1 bar using Langevin thermostat and barostat (38, 39), respectively. The SHAKE algorithm was used to constrain all bonds with hydrogen atoms (40). For the calculation of van der Waals interactions, a pairlist distance of 13.5 Å, a switching distance of 12 Å, and a cutoff of 10 Å were used. The Particle mesh Ewald (PME) method under periodic boundary conditions was utilized for the calculation of electrostatic interactions and forces (41).

The system was equilibrated in 6 steps. In the first step, protein backbone and side chain heavy atoms were restrained using harmonic potentials with force constants of 10 and 5 kcal/mol/Å^2, respectively, while the system was minimized for 10,000 timesteps using the steepest descent algorithm, followed by 100 ps of MD simulation. The modeled loop regions in the spike head and the linker regions connecting the different domain in the stalk were excluded from the restraints in this step to allow their adjustment. The restraints on the protein were sequentially reduced by half in each subsequent equilibration step, and the system was further equilibrated for 2 ns in each step. Additionally, coordinates of the lipid head group heavy atoms were restrained along the z axis (membrane normal) in order to maintain the membrane thickness during the whole equilibration phase with decreasing force constants from an initial value of 5 to 0.1 kcal/mol/Å^2 over the 6 equilibration steps. The first 3 equilibration steps were carried out using a timestep of 1 fs and the subsequent steps and the production runs with a timestep of 2 fs. After the equilibration phase, all the restraints were removed, and a production run was carried out for 5 μs.

As a control, full-length spike without glycosylations and palmitoylations was similarly prepared, embedded in an ERGIC-like lipid bilayer, equilibrated, and then simulated for 5 μs. The starting structures for both the glycosylated and non-glycosylated spike simulation systems along with the trajectories are available at: https://usegalaxy.org/u/tianle_chen/h/spiketrajectory.

Analysis. We first evaluated the structural stability of the spike over the course of the MD simulations, specially in the modeled TM domain. The global conformational dynamics of the spike protein obtained from the simulations were characterized in terms of the bending and twisting motions of the spike head with respect to the different domains in the stalk region. Additionally, the potential role of glycosylation in modulating these motions was analyzed in terms of direct interactions with the lipid bilayer as well as contacts formed between the glycans at the interfaces of different domains and the lipids. Finally, we also investigated the potential effect of palmitoylations on SARS-CoV-2 spike in modulating the membrane curvature.

Stability of the spike domains. The internal RMSD of different spike domains was calculated by superimposing the trajectory with respect to the starting structure of the respective domain using C_α atoms. Furthermore, we characterized the stability of the modeled TM domain by calculating the following: i) TM tilt (described earlier for the evaluation of the TM domain models). ii) TM self-rotation which is calculated as the angle between the first principal axis of the moment of inertia of the heavy atoms in each TM monomer with respect to its equilibrated configuration, and iii) residue contact map between each TM monomer and the other two, calculated for Cα atoms and using a half-contact distance of 4 Å.

Global structural dynamics. The global conformational dynamics of the spike protein were characterized in terms of the orientational changes in the spike head with respect to the different stalk domains. For this, the trajectory was separately aligned using either the spike neck, the HR2 domain, or the TM domain, and the motion of the spike head was quantified with respect to

the superimposed region in terms of the i) head bend, i.e., bending motion of the spike head, ii) head twist, i.e., twisting motion of the spike head in the xy plane, and iii) head distance to the superimposed region. For the calculation of the angles, specific vector representations were introduced for different domains as described below (see Fig. S10). For the calculation of the head bend, the spike head was represented by a vector connecting the C_α atoms of the bottom and top residues of the central helices (i.e., residues 986 to 1,034), and the angle was calculated with respect to its initial position. For the calculation of the head twist, the top of the spike head was first approximated by a triangle with the C_α atoms of residues 146 in the 3 spike monomers as vertices. The rotation of this triangle (quantified as the angle of one of its sides with regard to the initial position) was used as the twist angle. The head distance was calculated between the centroid of the triangle described above and the C_α atom of the bottom residues of the different stalk domains used for superimposition (residue 1159 for the spike neck, residue 1204 for the HR2 domain, and residue 1236 for the TM domain).

Multiple sequence alignment of human coronaviruses. In order to analyze the sequence conservation of the individual spike domains, multiple sequence alignment was performed for the protein from different human coronaviruses (HCoVs). Currently a total of seven known HCoVs exist, namely, 229E and NL63 from the alpha subfamily of coronaviruses, and OC43, HKU1, MERS-CoV, SARS-CoV, and SARS-CoV-2, from the beta subfamily. Additionally, the bat coronavirus RaTG13, a closely related homolog of SARS-CoV-2, was also included in the sequence alignment. Multiple sequence alignment for the above eight sequences was carried out using the MAFFT program with the L-INS-i method (42) and visualized using Jalview (43).

Isosurface and solvent accessible surface area calculations for glycans. In order to visualize the average shielding effect of the glycans around each part of the spike, the mass density map of the glycan molecules was quantified with the `VolMap` plugin in VMD. For each domain atomic density distribution of the glycan molecules was calculated after the molecular system was aligned using the protein domain of interest (e.g., spike head, spike neck and HR2 domain). The density maps of the individual domains were combined to obtain an average spatial glycan density. Density isosurfaces were then used to delineate the highly shielded regions on the surface of the spike domains. To quantify the shielding effect of the glycans on the binding of small molecules and peptides to the spike, we also measured the solvent accessible surface area (SASA) for both the glycosylated and non-glycosylated spike systems with probe radii ranging from 1.0 to 7.0 Å. The SASA was calculated with the Shrake-Rupley algorithm (44) which counts the number of solvent accessible mesh points. The distance between the mesh points was determined from the radii of the probes.

Glycan-lipid and glycan-glycan interactions. As the glycan molecules attached to the HR2 domain are situated close to the membrane and to the glycans on the neck region, their possible interactions were further analyzed. For this, we identified all heavy atom contacts between glycans and other glycans or lipids (cutoff of 4.5 Å). The number of glycan heavy-atom contacts in each monomer for each frame in the trajectory was denoted as the glycan contact number, calculated separately for glycan-glycan and glycan-lipid interactions.

Additionally, the bending of the HR2 domain with respect to the membrane as well as the angle between the neck region and the HR2 domain was also quantified. For the former, the trajectory was first aligned by rotating the first principle axes of the membrane patch to that in the first frame so that the membrane normal vector lied in the direction of the z. The HR2 bending was then calculated as the angle between the third principal axis of the HR2 domain, determined using the C_α atoms, and the z axis representing the membrane normal. The neck-HR2 bending was determined similarly by obtaining the angle between the third principal axis of the HR2 domain and the neck region.

Correlated motions in the spike. In order to identify potential correlations in the motions of the different domains of the spike, the Gram-Schmidt orthogonalization process was carried out, a widely used mathematical method to orthonormalize a set of vectors, in this case vectors representing orientations of the spike head, the HR2 domain, and the TM domain, generating 3 linearly independent, perpendicular vectors. For given vectors $\vec{v}_1$ (HR2), $\vec{v}_2$ (TM) and $\vec{v}_3$ (spike head), representing the C_α atoms of the HR2, TM and the central helices of the spike head, respectively, we define a plane formed by two vectors, e.g., $\vec{v}_1$ and $\vec{v}_2$, and obtain the components of $\vec{v}_3$ on this plane and a perpendicular plane by performing orthogonalization. Thus, the spike head's coplanar and orthogonal motions with respect to the HR2-TM domain bending can be derived to investigate the inter-correlation between different modes of bending.

Calculation of membrane curvature. Since the palmitoyl tails have been shown to contribute to the bending of the membrane in other viruses (45), the curvature of the membrane around the fully-palmitoylated spike system was examined. Briefly, the coordinates of the upper and lower leaflet headgroups were fitted with a trigonometric surface function by the least square method, to maintain the periodic condition, followed by the numerical calculation of the mean curvature for the fitted grid points. For comparison, the membrane curvature of the non-palmitoylated spike system was also reported.

Estimation of uncertainty. In order to calculate the uncertainties associated with the different quantities reported here, the single 5 μs MD trajectory was divided into 500 ns trajectories. Spearman's coefficient were calculated by first taking averaged angle and contacts within a short interval (10 ns) as sampling data points and then randomly selecting $\frac{1}{10}$ of all data points 10 times to calculate the mean coefficients and their standard deviations.

Supplementary Figures

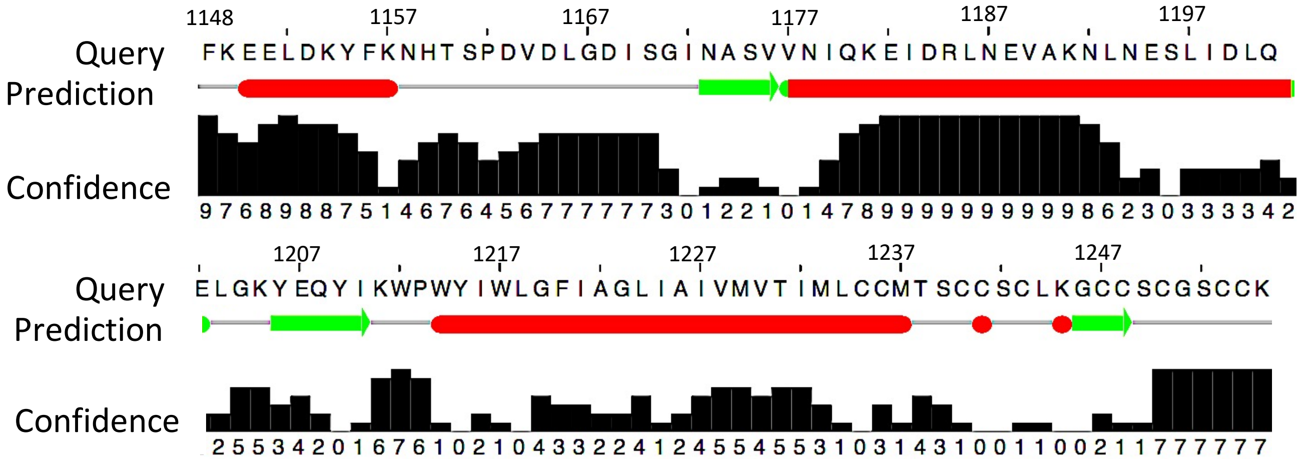

Fig. S1. Secondary structure prediction for the unresolved stalk in spike generated using JPred4 (13). Red bars represent predicted helical structures, and green bars represent $\beta-$sheets. The confidence level for the predictions ranges between 0 (low confidence) and 9 (high confidence).

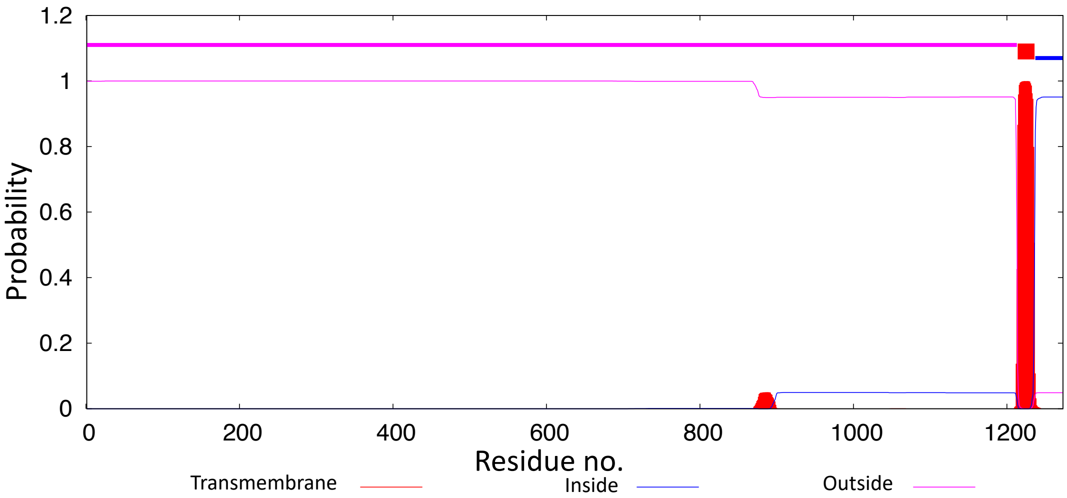

Fig. S2. Spike TM domain prediction using TMHMM (17). The plot shows the predicted probabilities of transmembrane as well as intracellular and extracellular regions of the protein sequence. At the top of the plot (between 1.0 and 1.2) the most probable location of the protein sequence (described in the bottom legend) is shown.

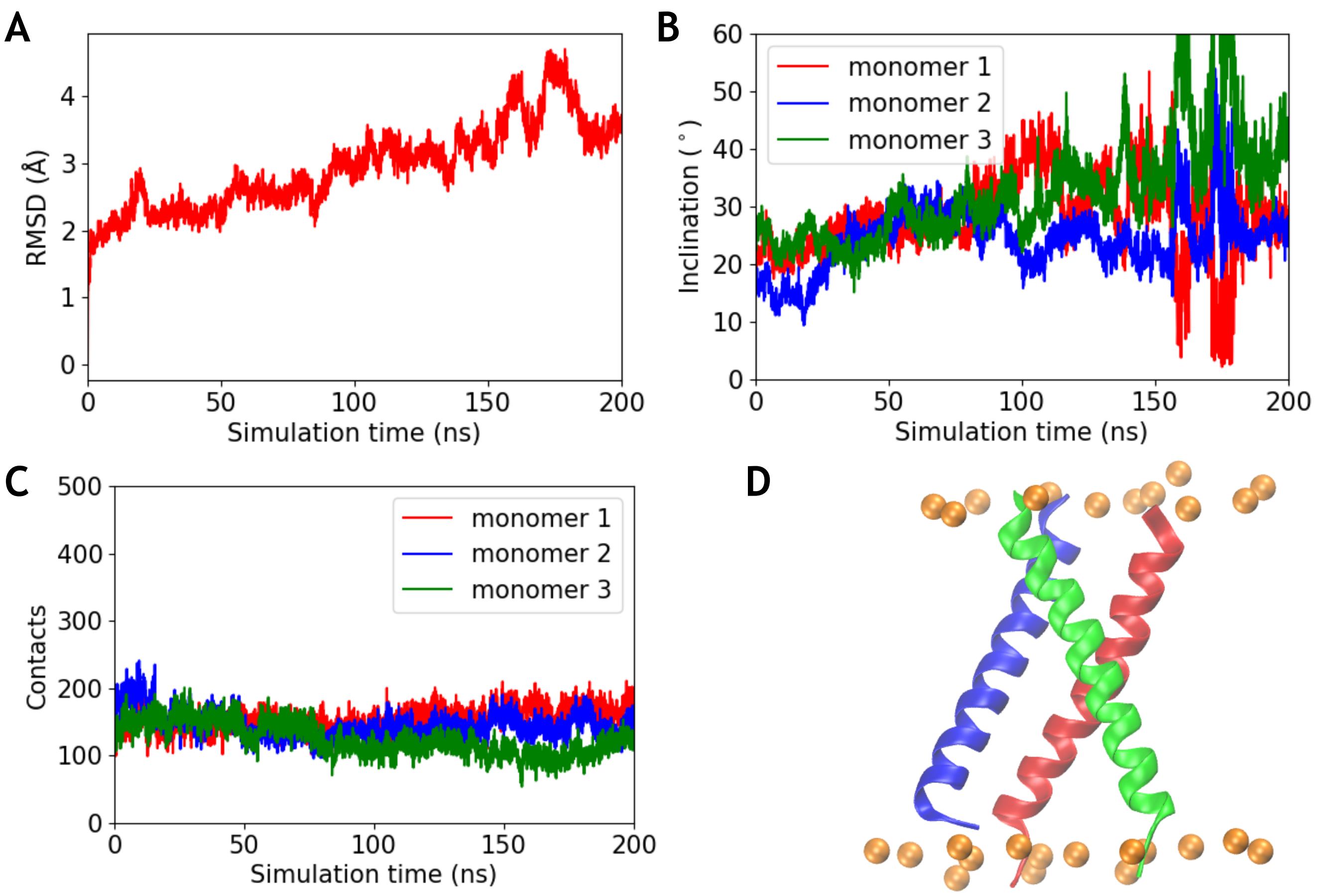

Fig. S3. Stability of predicted TM domain model 1. (A) RMSD of the simulated TM domain model 1 (refer to Table S3), after aligning The trajectory with the starting structure using C_α atoms. (B) The inclination of each TM monomer calculated with respect to the whole TM domain during the simulation. (C) The number of contacts between one TM monomer and the other two monomers in the TM trimer, quantified in terms of the coordination number (described in methods). (D) Snapshot of the TM domain structure at the end of the simulation is shown. The TM monomers are shown in same colors used in (B) and (C), and the phosphorus atoms of the lipid bilayer are shown as orange spheres.

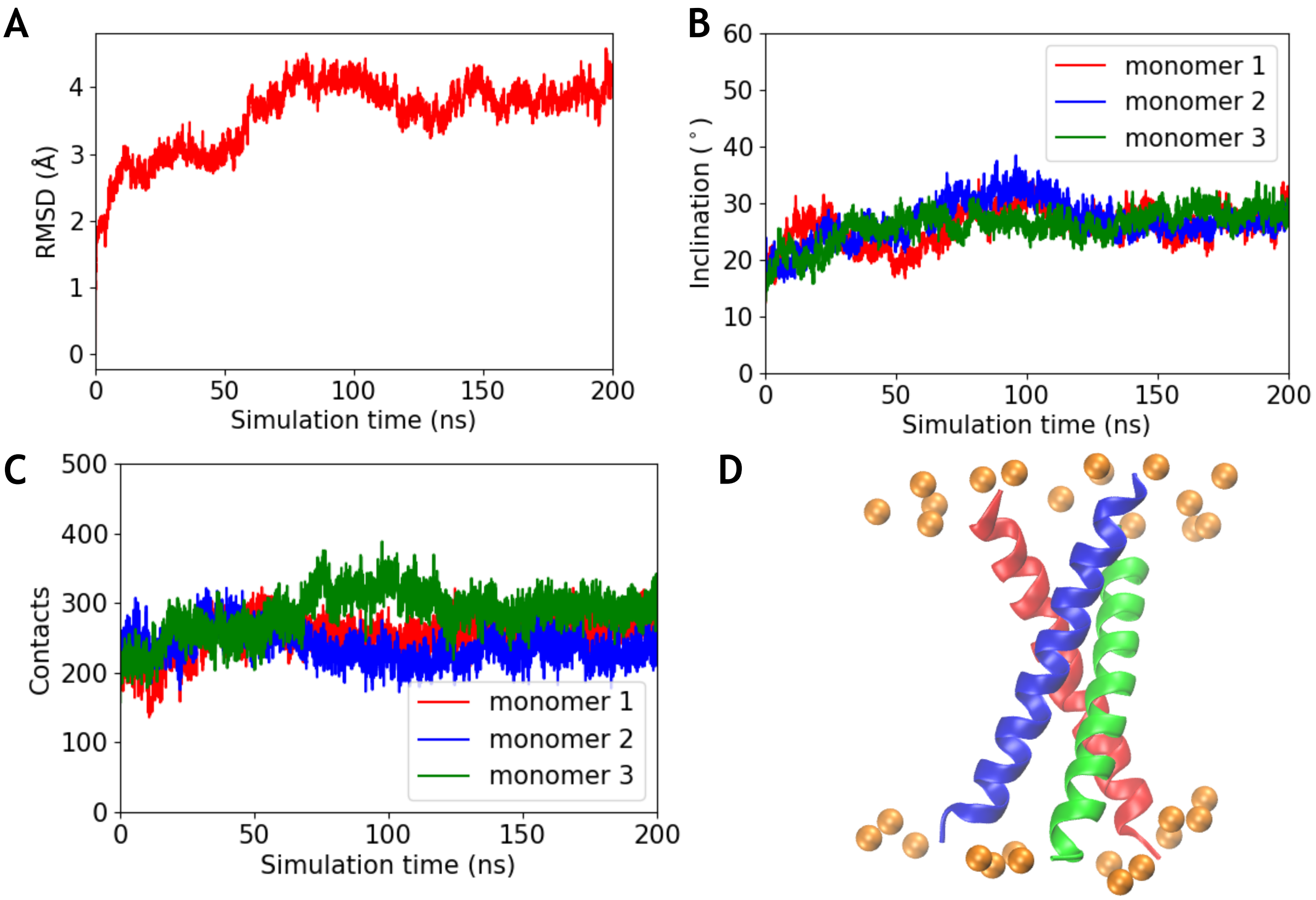

Fig. S4. Stability of predicted TM domain model 3. (A) RMSD of the simulated TM domain model 3 (refer to Table S3), after aligning The trajectory with the starting structure using C_α atoms. (B) The inclination of each TM monomer calculated with respect to the whole TM domain during the simulation. (C) The number of contacts between one TM monomer and the other two monomers in the TM trimer, quantified in terms of the coordination number (described in methods). (D) Snapshot of the TM domain structure at the end of the simulation is shown. The TM monomers are shown in same colors used in (B) and (C), and the phosphorus atoms of the lipid bilayer are shown as orange spheres.

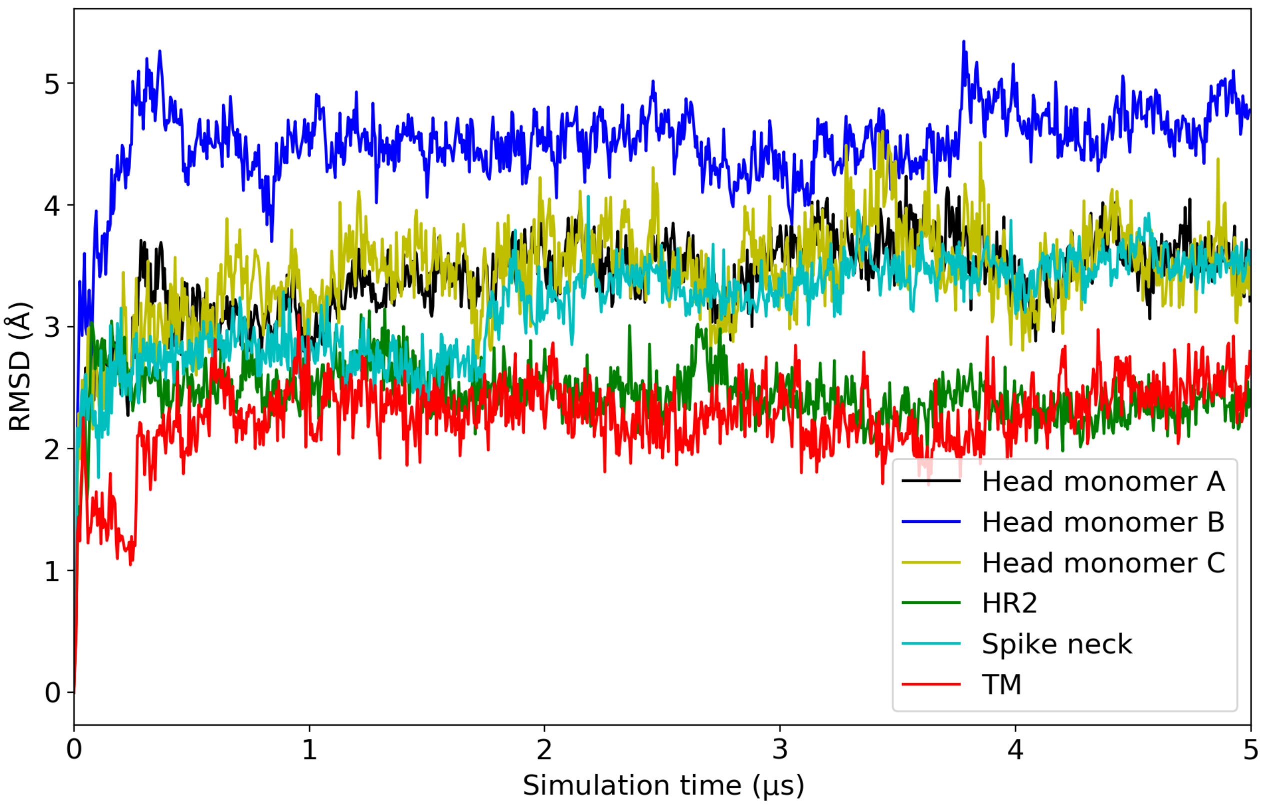

Fig. S5. C_α **RMSD of different spike domains:** The RMSD is calculated individually for different stalk domains: trimeric spike neck (residues 1139-1158), HR2 (residues 1173-1200), and the TM (residues 1213-1238), as well as the individual spike head monomers (monomer A, B and C (residues 1-1134). Monomer B of the spike head contains the RBD in the "up" configuration and is relatively more mobile compared to the other monomers containing RBD in the "down" configuration.

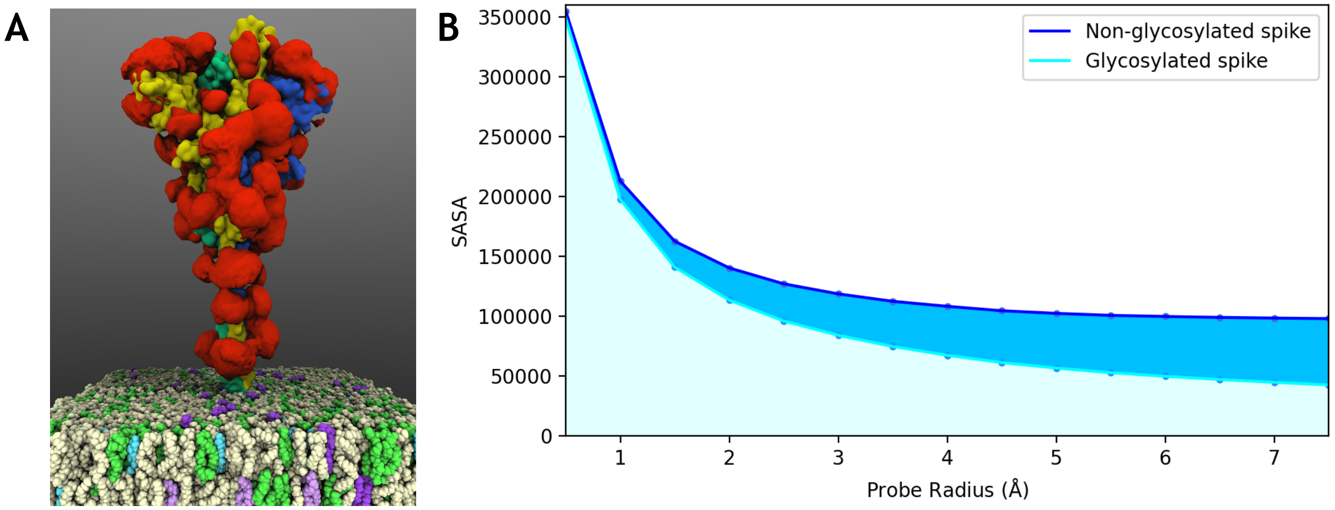

Fig. S6. Shielding effect of glycosylation. (A) Isosurface of the glycan density obtained from the MD simulation around the spike is shown. (B) SASA values of the glycosylated and non-glycosylated spike are shown at different probe radii. The SASA differences between the two structures is represented by the space between two curves.

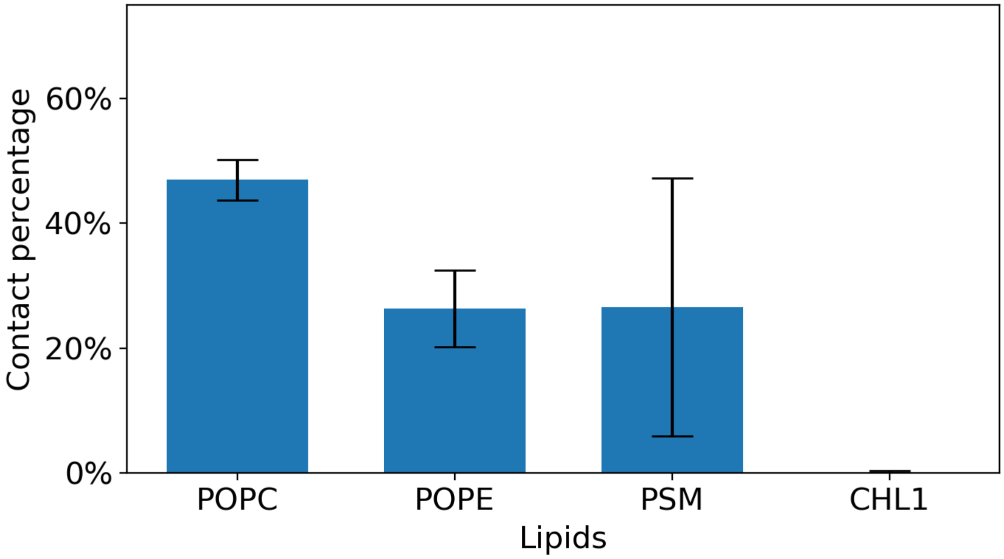

Fig. S7. Lipid-glycan contacts with different lipid types. The contact frequency of different lipid types with the glycans at N1198 is shown. Only frames with lipid-glycan contacts are included in the data. The reported percentages are normalized by the abundance of POPC, POPE, PSM, and cholesterol in the leaflet proximal to the glycans: POPC: POPE: PSM = 59.0%: 25.5%: 0.05%. The error bars are calculated by dividing the frames into 10 isometric pieces.

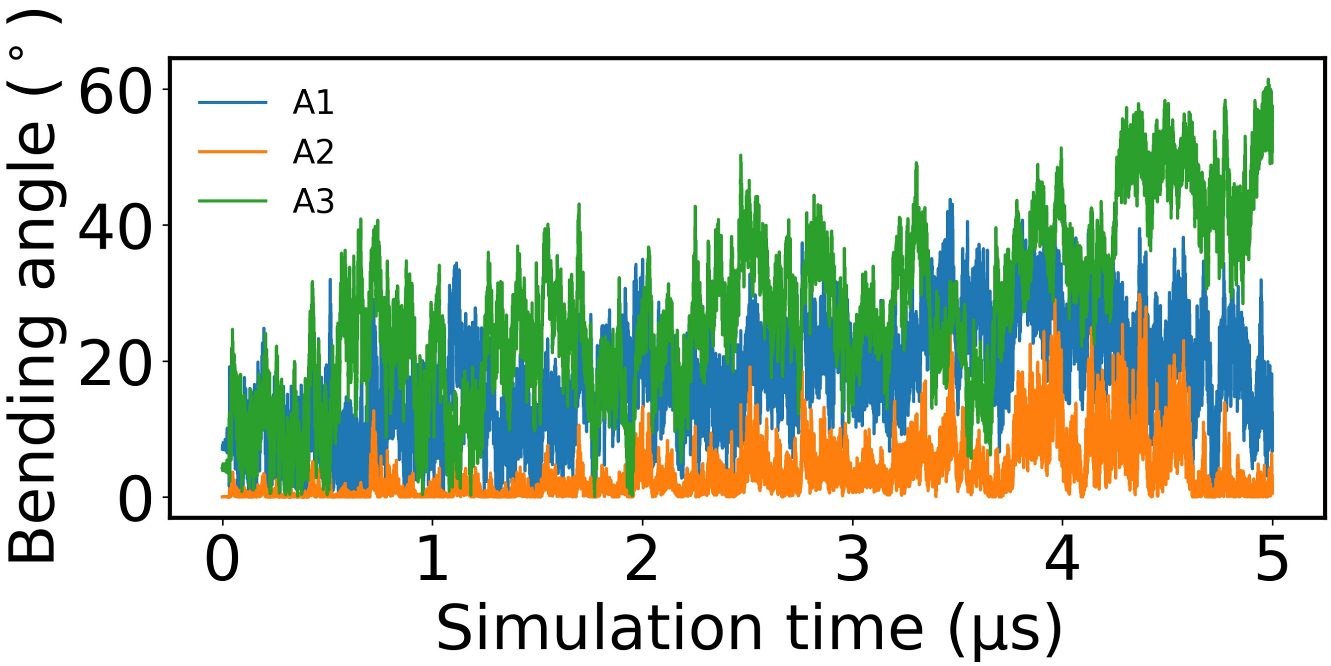

Fig. S8. Correlation of the global motions in non-glycosylated spike. The time series of the A1, A2, and A3 angles depicted in Fig. 6A are shown for the simulation of the non-glycosylated spike.

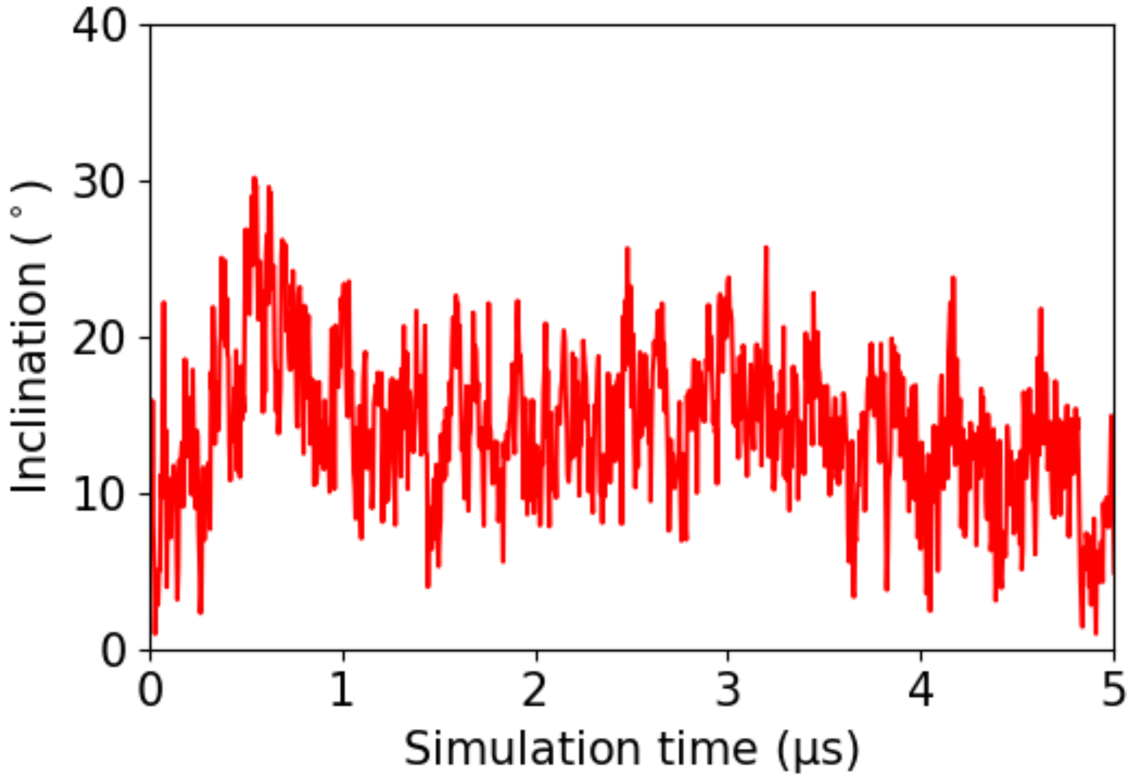

Fig. S9. Tilt angle of the TM domain in the membrane. The tilt angle of the TM domain is calculated with respect to its initial orientation. The angle was measured between the third principal component of inertia calculated using all heavy atoms of the TM domain with respect to that for the starting structure.

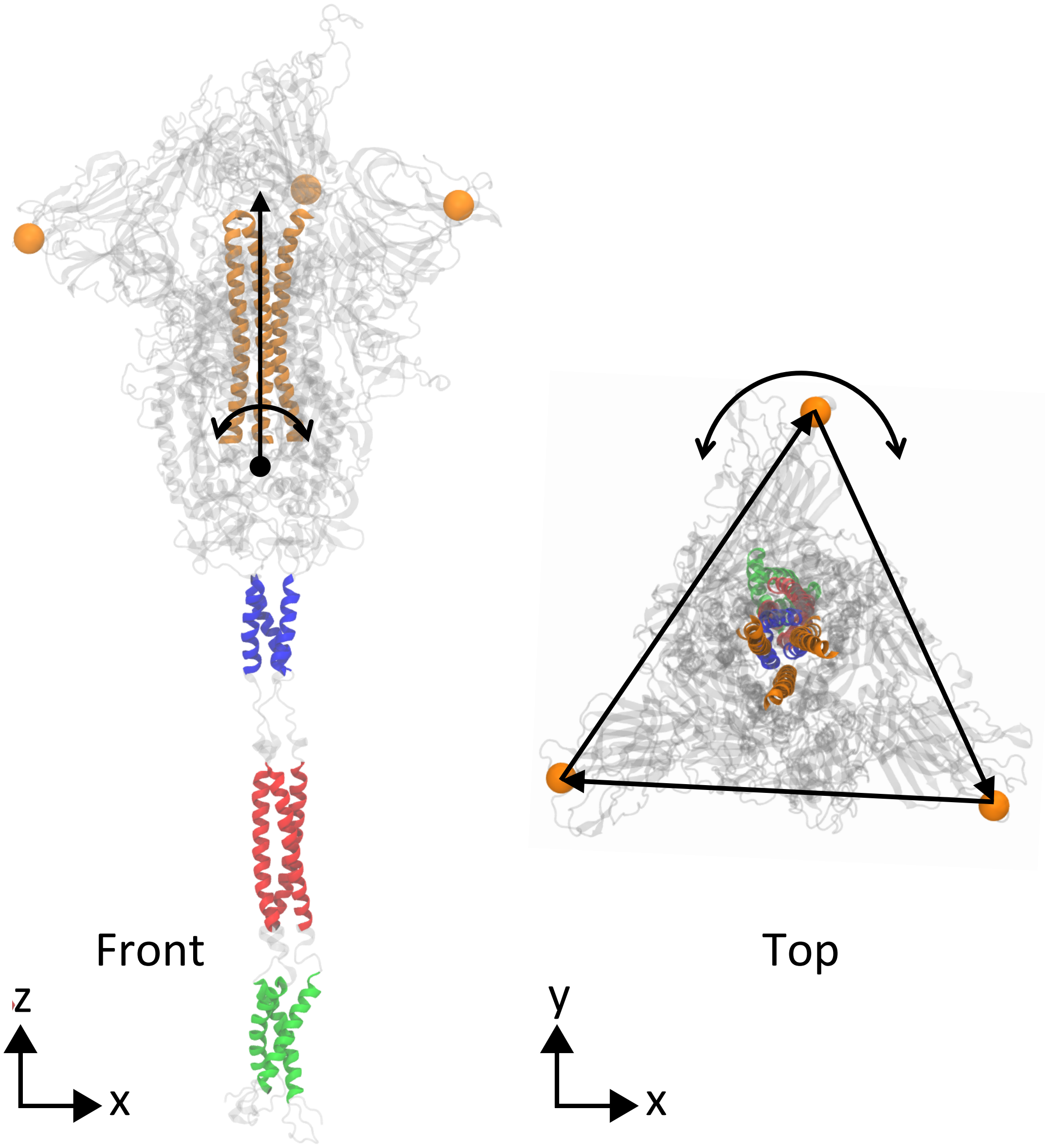

Fig. S10. Selection for global analysis. Front and top views of spike describing selections used to represent the different domains in the calculation of respective vectors. TM: green, HR2: red, spike neck: blue, and spike head (represented by central helices): orange. For the calculation of the head twist, the top of the spike was approximated by a triangle formed by the C_α atom of residue 146 from each monomer (orange vdW sphere). The sides of this triangle are the vectors used to calculate the bending and twist angles with respect to the starting geometry.

Supplementary Tables

Table S1. Lipid composition of the simulated membranes. The lipid composition (%) of the membrane patch representing the ERGIC lipid composition.

Leflet	POPC	POPE	Cholesterol	POPS	POPI	PSM
Outer	59.0	25.5	15.0	0.0	0.0	0.5
Inner	45.0	18.5	15.0	6.0	15.0	0.5

Site	Composition and type	Sequence
N61	HexNAc(2)Hex(5) M5	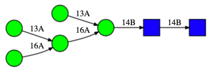
N122		
N603		
N709		
N717		
N801		
N1074		
N234	HexNAc(2)Hex(8) M8	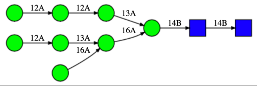
N657	HexNAc(3)Hex(6) Hybrid	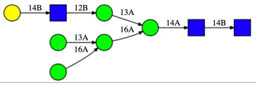
N149	HexNAc(4)Hex(3) Fuc(1) Complex	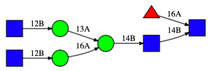
N331		
N343		
N616		
N1134		
N1158	HexNAc(4)Hex(4) Complex	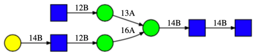
N1098	HexNAc(4)Hex(4) Fuc(1)NeuAc(1) Complex	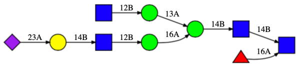
N165	HexNAc(4)Hex(5) Fuc(3)NeuAc(1) Complex	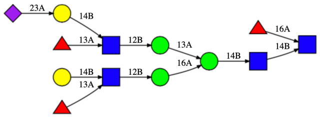
N282	HexNAc(5)Hex(3) Fuc(1) Complex	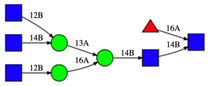
N17	HexNAc(5)Hex(4) Fuc(1) Complex	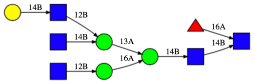
N1173	HexNAc(6)Hex(3) Fuc(1) Complex	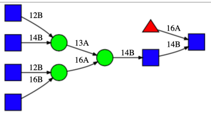
N74	HexNAc(6)Hex(4) Fuc(1)NeuAc(1) Complex	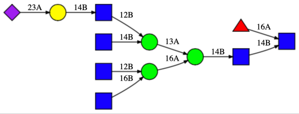
N1194	HexNAc(6)Hex(5) Fuc(1)NeuAc(1) Complex	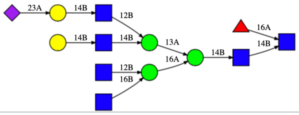
T323	O-glycan	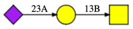

Table S2. Glycosylation sites and types. The glycosylation sites in spike, as well as the respective composition of the sugar moiety. The sequence graph shown for the glycans follows the standard of Symbol Nomenclature For Glycans (SNFG) (46).

	Model	Cluster	Score		Model	Cluster	Score
**1**	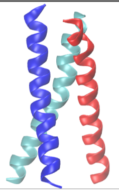	183	-635.1	**6**	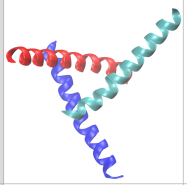	60	-638.2
**2**	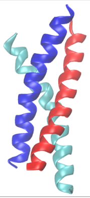	152	-611.6	**7**	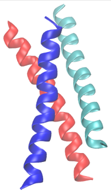	41	-599.5
**3**	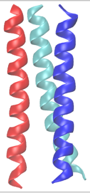	134	-604.7	**8**	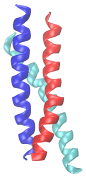	37	-594.2
**4**	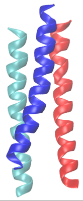	76	-642.2	**9**	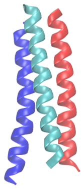	20	-612.0
**5**	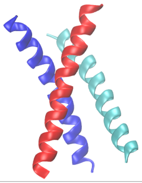	69	-595.1	**10**	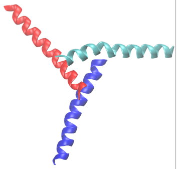	19	-646.9

Table S3. TM domain trimer models predicted by Cluspro. The predicted structures of the TM domain trimers as predicted by Cluspro (23) are shown. The 'cluster' column denotes the cluster size, which is the respective number of models present in each cluster after clustering is carried out on the selected TM domain trimer models. The 'score' shows the predicted binding energy calculated using the energy function of Cluspro.

Supplementary Movie Captions

Movie S1. The overview of the full spike system embedded in ERGIC membrane and the global motions of the spike head with respect to the stalk during the simulations are shown. Each spike monomer and constituent membrane lipids are shown in different colors and the glycans are shown in red.

Movie S2. The interactions between the glycans in the HR2 domain and lipids along with the bending of HR2 domain towards the membrane are shown in the movie. The glycans at the bottom of HR2 domain are shown in the same color as each of the spike monomer, respectively.

Movie S3. The contacts between glycans at the HR2 domain and neck interface, and the bending of neck region towards HR2 are shown in the movie. The glycans at the top of HR2 domain are shown in the same color as each of the monomer.

Movie S4. The correlated motions between the different domains of the spike are shown. The trajectory is superimposed with respect to the membrane. HR2 domain is represented by a transparent surface (green) overlayed on top of the structure.

References

1. LK Tamm, X Han, Viral fusion peptides: a tool set to disrupt and connect biological membranes. *Biosci. Rep.* **20**, 501–518 (2000).
2. JM White, SE Delos, M Brecher, K Schornberg, Structures and mechanisms of viral membrane fusion proteins: multiple variations on a common theme. *Crit. Rev. Biochem. Mol. Biol.* **43**, 189–219 (2008).
3. AC Walls, et al., Structure, function, and antigenicity of the SARS-CoV-2 spike glycoprotein. *Cell* **181**, 281–292 (2020).
4. Y Yuan, et al., Cryo-EM structures of MERS-CoV and SARS-CoV spike glycoproteins reveal the dynamic receptor binding domains. *Nat. Commun.* **8**, 1–9 (2017).
5. M Gui, et al., Cryo-electron microscopy structures of the SARS-CoV spike glycoprotein reveal a prerequisite conformational state for receptor binding. *Cell Res.* **27**, 119–129 (2017).
6. RN Kirchdoerfer, et al., Stabilized coronavirus spikes are resistant to conformational changes induced by receptor recognition or proteolysis. *Sci. Rep.* **8**, 1–11 (2018).
7. YJ Park, et al., Structures of MERS-CoV spike glycoprotein in complex with sialoside attachment receptors. *Nat. Struct. Mol. Biol.* **26**, 1151–1157 (2019).
8. R Yan, et al., Structural basis for the recognition of SARS-CoV-2 by full-length human ACE2. *Science* **367**, 1444–1448 (2020).
9. S Vilar, G Cozza, S Moro, Medicinal chemistry and the molecular operating environment (MOE): application of QSAR and molecular docking to drug discovery. *Curr. Top. Med. Chem.* **8**, 1555–1572 (2008).
10. S Raman, et al., Structure prediction for CASP8 with all-atom refinement using Rosetta. *Proteins: Struct., Func., Bioinf.* **77**, 89–99 (2009).
11. Y Song, et al., High-resolution comparative modeling with RosettaCM. *Structure* **21**, 1735–1742 (2013).
12. S Hakansson-McReynolds, S Jiang, L Rong, M Caffrey, Solution structure of the severe acute respiratory syndrome-coronavirus heptad repeat 2 domain in the prefusion state. *J. Biol. Chem.* **281**, 11965–11971 (2006).
13. A Drozdetskiy, C Cole, J Procter, GJ Barton, JPred4: a protein secondary structure prediction server. *Nucleic Acids Res.* **43**, W389–W394 (2015).
14. R Broer, B Boson, W Spaan, FL Cosset, J Corver, Important role for the transmembrane domain of severe acute respiratory syndrome coronavirus spike protein during entry. *J. Virol.* **80**, 1302–1310 (2006).
15. BJ Bosch, CA de Haan, SL Smits, PJ Rottier, Spike protein assembly into the coronavirion: exploring the limits of its sequence requirements. *Virology* **334**, 306–318 (2005).
16. J Dev, et al., Structural basis for membrane anchoring of HIV-1 envelope spike. *Science* **353**, 172–175 (2016).
17. A Krogh, B Larsson, G von Heijne, EL Sonnhammer, Predicting transmembrane protein topology with a hidden markov model: application to complete genomes. *J. Mol. Biol.* **305**, 567–580 (2001).
18. S Jo, T Kim, VG Iyer, W Im, CHARMM-GUI: a web-based graphical user interface for CHARMM. *J. Comput. Chem.* **29**, 1859–1865 (2008).
19. E Wu, et al., CHARMM-GUI membrane builder toward realistic biological membrane simulations. *J. Comput. Chem.* **35**, 1997–2004 (2014).
20. J Krijnse-Locker, M Ericsson, P Rottier, G Griffiths, Characterization of the budding compartment of mouse hepatitis virus: evidence that transport from the RER to the Golgi complex requires only one vesicular transport step. *J. Cell Biol.* **124**, 55–70 (1994).
21. A Schweizer, H Clausen, G Van Meer, HP Hauri, Localization of O-glycan initiation, sphingomyelin synthesis, and glucosylceramide synthesis in Vero cells with respect to the endoplasmic reticulum-Golgi intermediate compartment. *J. Biol. Chem.* **269**, 4035–4041 (1994).

22. BG Hogue, CE Machamer, Coronavirus structural proteins and virus assembly in *Nidoviruses.* (American Society of Microbiology), pp. 179–200 (2008).
23. D Kozakov, et al., The ClusPro web server for protein-protein docking. *Nat. Protoc.* **12**, 255–278 (2017).
24. G Fiorin, ML Klein, J Hénin, Using collective variables to drive molecular dynamics simulations. *Mol. Phys.* **111**, 3345–3362 (2013).
25. EA Coutsias, C Seok, KA Dill, Using quaternions to calculate RMSD. *J. Chem. Phys.* **25**, 1849–1857 (2004).
26. W Humphrey, A Dalke, K Schulten, VMD: Visual molecular dynamics. *J. Mol. Graph.* **14**, 33–38 (1996).
27. A Fiser, RKG Do, A Šali, Modeling of loops in protein structures. *Prot. Sci.* **9**, 1753–1773 (2000).
28. Y Watanabe, JD Allen, D Wrapp, JS McLellan, M Crispin, Site-specific glycan analysis of the SARS-CoV-2 spike. *Science* **369**, 330–333 (2020).
29. A Shajahan, NT Supekar, AS Gleinich, P Azadi, Deducing the N-and O-glycosylation profile of the spike protein of novel coronavirus SARS-CoV-2. *Glycobiology* **30**, 981–988 (2020).
30. CM Petit, et al., Palmitoylation of the cysteine-rich endodomain of the SARS–coronavirus spike glycoprotein is important for spike-mediated cell fusion. *Virology* **360**, 264–274 (2007).
31. CM Petit, et al., Palmitoylation of the cysteine-rich endodomain of the SARS–coronavirus spike glycoprotein is important for spike-mediated cell fusion. *Virology* **360**, 264 – 274 (2007).
32. H Woo, et al., Developing a fully glycosylated full-length SARS-CoV-2 spike protein model in a viral membrane. *J. Phys. Chem. B* **124**, 7128–7137 (2020).
33. SJ Park, et al., CHARMM-GUI Glycan Modeler for modeling and simulation of carbohydrates and glycoconjugates. *Glycobiology* **29**, 320–331 (2019).
34. JC Phillips, et al., Scalable molecular dynamics with NAMD. *J. Comput. Chem.* **26**, 1781–1802 (2005).
35. JC Phillips, et al., Scalable molecular dynamics on CPU and GPU architectures with NAMD. *J. Chem. Phys.* **153**, 044130 (2020).
36. K Hart, et al., Optimization of the CHARMM additive force field for DNA: Improved treatment of the BI/BII conformational equilibrium. *J. Chem. Theory Comput.* **8**, 348–362 (2012).
37. JB Klauda, et al., Update of the CHARMM all-atom additive force field for lipids: Validation on six lipid types. *J. Phys. Chem. B* **114**, 7830–7843 (2010).
38. GJ Martyna, DJ Tobias, ML Klein, Constant pressure molecular dynamics algorithms. *J. Chem. Phys.* **101**, 4177–4189 (1994).
39. SE Feller, Y Zhang, RW Pastor, Constant pressure molecular dynamics simulation: the Langevin piston method. *J. Chem. Phys.* **103**, 4613–4621 (1995).
40. JP Ryckaert, G Ciccotti, HJC Berendsen, Numerical integration of the Cartesian equations of motion of a system with constraints: Molecular dynamics of *n*-alkanes. *J. Comp. Phys.* **23**, 327–341 (1977).
41. T Darden, D York, L Pedersen, Particle mesh Ewald: an $N \cdot \log(N)$ method for Ewald sums in large systems. *J. Chem. Phys.* **98**, 10089–10092 (1993).
42. K Katoh, K Misawa, K Kuma, T Miyata, MAFFT: a novel method for rapid multiple sequence alignment based on fast fourier transform. *Nucleic Acids Res.* **30**, 3059–3066 (2002).
43. M Clamp, J Cuff, SM Searle, GJ Barton, The Jalview Java alignment editor. *Bioinformatics* **20**, 426–427 (2004).
44. A Shrake, JA Rupley, Environment and exposure to solvent of protein atoms. lysozyme and insulin. *J. Mol. Biol.* **79**, 351–371 (1973).
45. P Chlanda, et al., Palmitoylation contributes to membrane curvature in influenza A virus assembly and hemagglutinin-mediated membrane fusion. *J. Virol.* **91**, e00947–17 (2017).
46. A Varki, et al., Symbol nomenclature for graphical representations of glycans. *Glycobiology* **25**, 1323–1324 (2015).
